# One-step ethylene production from a four-component gas mixture by a single physisorbent

**DOI:** 10.1038/s41467-021-26473-8

**Published:** 2021-11-11

**Authors:** Jian-Wei Cao, Soumya Mukherjee, Tony Pham, Yu Wang, Teng Wang, Tao Zhang, Xue Jiang, Hui-Juan Tang, Katherine A. Forrest, Brian Space, Michael J. Zaworotko, Kai-Jie Chen

**Affiliations:** 1 Key Laboratory of Special Functional and Smart Polymer Materials of Ministry of Industry and Information Technology, Xi’an Key Laboratory of Functional Organic Porous Materials, School of Chemistry and Chemical Engineering, Northwestern Polytechnical University, Xi’an, Shaanxi PR China; 2 Bernal Institute, Department of Chemical Sciences, University of Limerick, Limerick, Republic of Ireland; 3 Department of Chemistry, Technical University of Munich, Garching b, München Germany; 4 Department of Chemistry, University of South Florida, Tampa, FL USA; 5 Department of Chemistry, North Carolina State University, Raleigh, USA

**Keywords:** Metal-organic frameworks, Porous materials

## Abstract

One-step adsorptive purification of ethylene (C_2_H_4_) from four-component gas mixtures comprising acetylene (C_2_H_2_), ethylene (C_2_H_4_), ethane (C_2_H_6_) and carbon dioxide (CO_2_) is an unmet challenge in the area of commodity purification. Herein, we report that the ultramicroporous sorbent Zn-atz-oba (H_2_oba = 4,4-dicarboxyl diphenyl ether; Hatz = 3-amino-1,2,4-triazole) enables selective adsorption of C_2_H_2_, C_2_H_6_ and CO_2_ over C_2_H_4_ thanks to the binding sites that lie in its undulating pores. Molecular simulations provide insight into the binding sites in Zn-atz-oba that are responsible for coadsorption of C_2_H_2_, C_2_H_6_ and CO_2_ over C_2_H_4_. Dynamic breakthrough experiments demonstrate that the selective binding exhibited by Zn-atz-oba can produce polymer-grade purity (>99.95%) C_2_H_4_ from binary (1:1 for C_2_H_4_/C_2_H_6_), ternary (1:1:1 for C_2_H_2_/C_2_H_4_/C_2_H_6_) and quaternary (1:1:1:1 for C_2_H_2_/C_2_H_4_/C_2_H_6_/CO_2_) gas mixtures in a single step.

## Abstract (web summary)

The purification of ethylene is an industrially relevant process. Here, the authors report the one-step separation of ethylene from quaternary gas mixtures of hydrocarbons and CO_2_ using a single metal–organic framework-based physisorbent.

## Introduction

Ethylene (C_2_H_4_) is a feedstock for the production of plastics, detergents and coatings and its production, now approaching 200 million tons per year, continues to grow^1^. The energy footprints for purification of C_2_H_4_ and propylene (C_3_H_6_), also one of the highest volume products of the chemical industry, account for *ca*. 0.3% of the global energy demand^2^. The presence of impurities is a consequence of the steam pyrolysis process used to produce C_2_H_4_, which in turn results in acetylene (C_2_H_2_), carbon dioxide (CO_2_), ethane (C_2_H_6_) and other downstream products including propylene (C_3_H_6_), propane (C_3_H_8_), hydrogen (H_2_), C4 and higher light hydrocarbons^3,4^, which are easily separated by the difference of boiling points. Polymer-grade (>99.95% purity) C_2_H_4_ is generated by stepwise removal in downstream purification processes: CO_2_ is removed using caustic soda; C_2_H_2_ is eliminated via catalytic hydrogenation with noble-metal catalysts at high temperature and pressure; cryogenic distillation is typically employed to remove C_2_H_6_^5^.

**Fig. 1:**
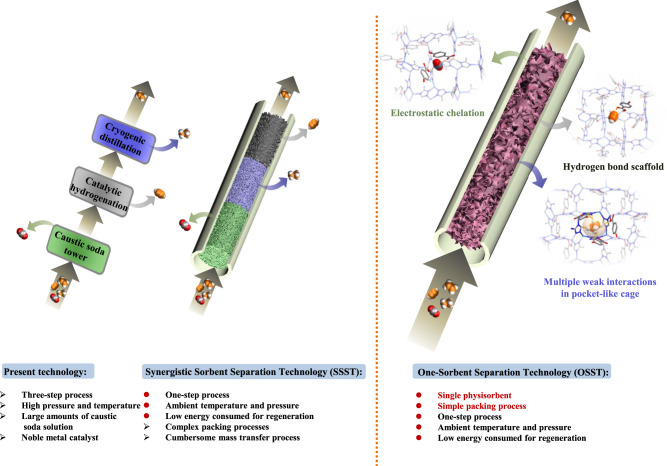
Comparison of ethylene purification technologies. State-of-the-art separation technology is compared to synergistic sorbent separation technology (SSST) and “one-sorbent separation technology”, OSST.

To mitigate the high energy footprint of C_2_H_4_ production, researchers have typically focused upon the development of a new generation of physisorbents that exhibit affinity for one gas over others in a gas mixture. The promise of physisorbents lies with their relatively low energy consumption compared to distillation processes thanks to facile regeneration/recycling^6^. However, physisorbents tend to be unsuitable for ethylene purification because the kinetic diameter of C_2_H_4_ (4.1 Å) sits between CO_2_ (3.3 Å), C_2_H_2_ (3.3 Å) and C_2_H_6_ (4.4 Å), precluding the possibility of molecular sieving by pore size engineered physisorbents^7^. Since the quadrupole moment of C_2_H_4_ (1.5 × 10^−26^ esu cm^2^) also lies between CO_2_ (4.3 × 10^−26^ cm^2^), C_2_H_2_ (7.2 × 10^−26^ esu cm^2^) and C_2_H_6_ (0.65 × 10^−26^ esu cm^2^)^8^, one-step purification of C_2_H_4_ by thermodynamics (selective binding) has thus far proven to be elusive. Metal organic materials (MOMs)^9^, also called metal-organic frameworks (MOFs)^10,11^ or porous coordination polymers (PCPs)^12^, have promising applications as C2 and CO_2_ selective physisorbents for several binary mixtures, including C_2_H_2_/C_2_H_4_, C_2_H_4_/C_2_H_6_, C_2_H_6_/C_2_H_4_, C_2_H_2_/CO_2_ and CO_2_/C_2_H_2_^13–34^ There are also examples of physisorbents that are effective against ternary C2 and C2-CO_2_ mixtures such as C_2_H_2_/C_2_H_4_/C_2_H_6_ and C_2_H_2_/C_2_H_4_/CO_2_^35–41^. Certain classes of physisorbents are amenable to systematic fine-tuning of pore chemistry and pore size^42,43^ and have resulted in “second generation” sorbents with > one order of magnitude improvement in performance^18,24,44,45,47^. Nevertheless, we are aware of only one report of C_2_H_4_ purification from quaternary mixtures using physisorbents as reported by us in 2019 by introducing the synergistic sorbent separation technology (SSST)^46^ concept, which enables one-step production of high-purity ethylene from a quaternary C_2_H_2_-C_2_H_4_-C_2_H_6_-CO_2_ mixture by exploiting three benchmark sorbents, Zn-atz-ipa, SIFSIX-3-Ni, and TIFSIX-2-Cu-i. These sorbents were tandem-packed in a single column to sequentially remove C_2_H_6_, CO_2_, and C_2_H_2_, respectively (Fig. 1). Unfortunately, the interplay of packing sequence and gas mass transfer in SSST can make industrial-scale processes infeasible and, as illustrated in Fig. 1, a single sorbent that coadsorbs C_2_H_2_, C_2_H_6_, and CO_2_ would be desirable for quaternary gas separations such as the purification of C_2_H_4_ from a C_2_H_2_-C_2_H_4_-C_2_H_6_-CO_2_ mixture. Herein we report that the challenge of one-step C_2_H_4_ purification from a quaternary mixture (C_2_H_2_-C_2_H_4_-C_2_H_6_-CO_2_) is achieved by a single physisorbent, the ultramicroporous coordination network Zn-atz-oba.

## Results

### Synthesis and characterization of Zn-atz-oba

**Fig. 2:**
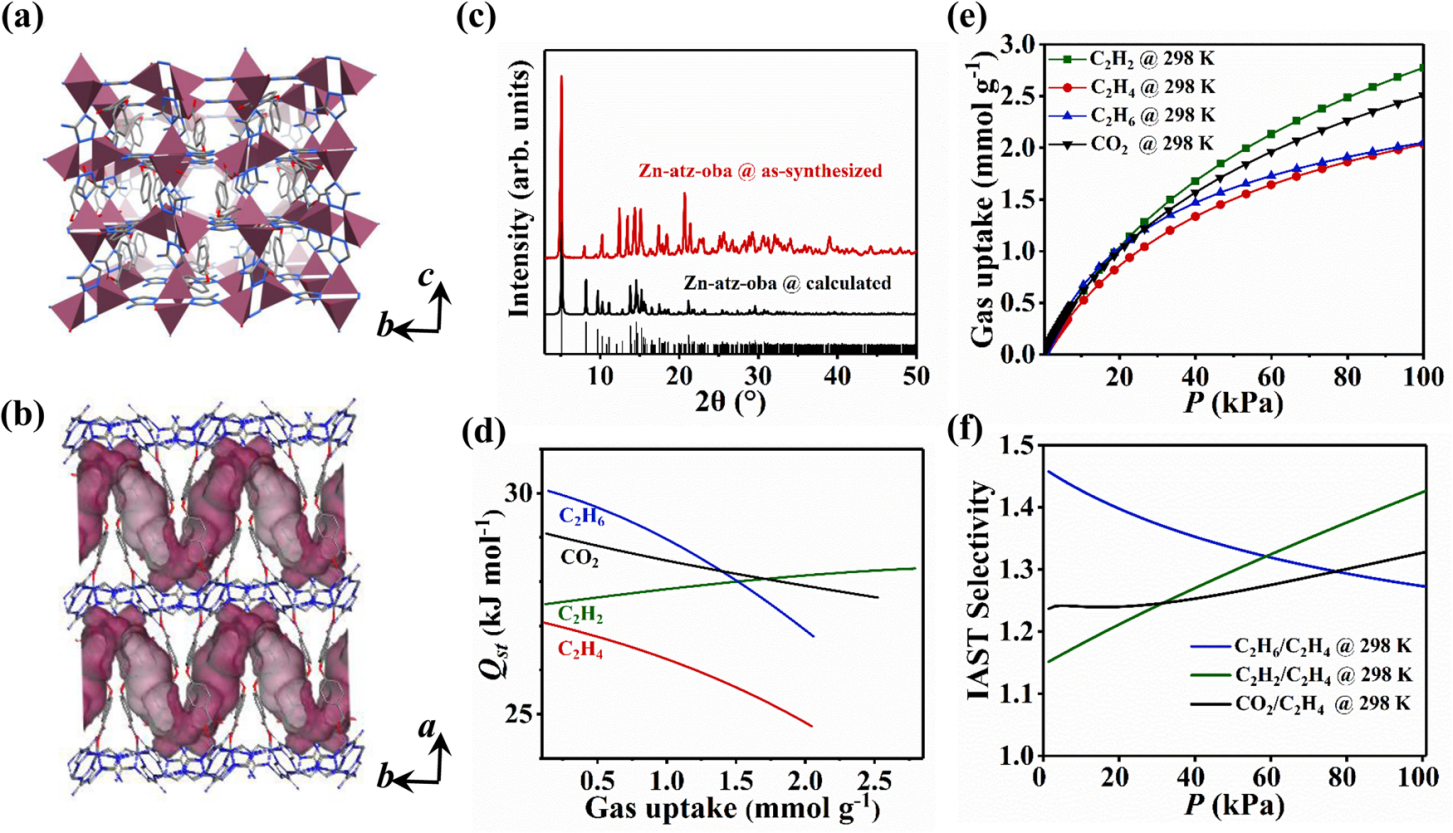
Pore structure and gas sorption properties of Zn-atz-oba. Views of (**a**) the pore structure and (**b**) the Connolly surface of Zn-atz-oba when viewed along the *a*- and *c*-axis, respectively. H-atoms are omitted for clarity. (**c**) PXRD patterns confirm the bulk phase purity of Zn-atz-oba. (**d**) Adsorption enthalpy profiles (*Q*_st_) for Zn-atz-oba. (**e**) Gas sorption isotherms of Zn-atz-oba at 298 K. (**f**) Binary 1:1 (v/v) IAST selectivity of Zn-atz-oba at 298 K. color codes for (**a**) and (**b**): carbon = gray; nitrogen = blue; oxygen = red; Zn polyhedra in (**a**) = tyrian purple; Connolly surface in (**b**) = purple.

Zn-atz-oba was synthesized following a previously reported protocol (for details see Methods section)^47^. The bulk phase purity of crystalline samples was confirmed by powder X-ray diffraction, PXRD (Fig. 2c). As shown in Fig. 2a, b, Zn(II) cations are linked by atz^−^ anions to form 2D undulating layers with dinuclear Zn(II) clusters as nodes. These layers are further cross-linked via oba^2−^ ligands to form a pcu topology network. As revealed by thermogravimetric analysis (Supplementary Fig. 1), activated Zn-atz-oba is fully desolvated and stable until ca. 673 K. As calculated by PLATON^48^, the void space of Zn-atz-oba is 35.9%.

The pore volume, Langmuir and Brunauer−Emmett−Teller (BET) specific surface area for Zn-atz-oba are 0.287 cm^3^ g^−1^, 783.1 m^2^ g^−1^, and 710.7 m^2^ g^−1^, respectively, as calculated from its cryogenic (77 K) N_2_ adsorption isotherm (Supplementary Figs. 2–4). Both parameters are in agreement with the crystal structure derived pore volume (0.283 cm^3^ g^−1^) and the Langmuir surface area calculated therefrom (755 m^2^ g^−1^), respectively. Horvath-Kawazoe model (pore geometry: slit) based pore size distribution analysis revealed the aperture distribution to be ultramicroporous between 0.32 and 0.44 nm (Supplementary Fig. 5).

### Adsorption isotherm, selectivity, and enthalpy

The C_2_H_2_, C_2_H_4_, C_2_H_6_, and CO_2_ adsorption isotherms of activated Zn-atz-oba were recorded at 298 and 273 K (Fig. 2e and Supplementary Fig. 6). Interestingly, C_2_H_2_, C_2_H_6_, and CO_2_ exhibited higher adsorption uptakes at 298 K across the entire pressure range tested, 0–100 kPa, especially in the low-pressure region, 0–20 kPa. These uptakes suggest selective adsorption of C_2_H_2_, CO_2_, and C_2_H_6_ over C_2_H_4_ by Zn-atz-oba. Low-coverage isosteric adsorption enthalpies were determined by virial fit of the isotherm data (see Methods section, Supplementary Note 2: Adsorption enthalpy calculation, and Supplementary Figs. 7–10) and the trends (Fig. 2d) correlate well with their low-pressure (until 20 kPa) saturation uptake capacities as follows: *Q*_st_(C_2_H_6_) (30.0 kJ mol^−1^) > *Q*_st_(CO_2_) (29.0 kJ mol^−1^) > *Q*_st_(C_2_H_2_) (27.5 kJ mol^−1^) > *Q*_st_(C_2_H_4_) (27.0 kJ mol^−1^). We also note that the adsorption enthalpies of all four gases are below 35 kJ mol^−1^, an indication that Zn-atz-oba should exhibit a relatively low energy footprint for regeneration^27,45^.

Adsorption selectivity is also a key indicator of separation performance. The adsorption selectivities of Zn-atz-oba for C_2_H_2_/C_2_H_4_, C_2_H_6_/C_2_H_4_, and CO_2_/C_2_H_4_ were calculated using Ideal Adsorbed Solution Theory^49^ (IAST) after fitting the single-component adsorption isotherms to the Langmuir-Freundlich model (Supplementary Figs. 11–14 and Supplementary Table 1). As shown in Fig. 2f, at 298 K and 100 kPa, the selectivities are 1.43 (C_2_H_2_/C_2_H_4_), 1.27 (C_2_H_6_/C_2_H_4_), and 1.33 (CO_2_/C_2_H_4_). C_2_H_6_/C_2_H_4_ and C_2_H_2_/C_2_H_4_ selectivities for Zn-atz-oba are comparable to the current benchmark sorbents that enable C_2_H_4_ purification from C_2_H_2_/C_2_H_4_/C_2_H_6_ 1:1:1 ternary mixtures (1.2 and 1.8 for TJT-100^35^, 1.46 and 1.09 for Azole-Th-1^36^, 1.32 and 1.4 for NPU-1^39^, 1.4 and 1.07 for UPC-612 and 1.5 and 1.4 for UPC-613^41^, respectively) (Supplementary Table 3). The selectivity for CO_2_/C_2_H_4_ is also comparable to that of C_2_H_6_/C_2_H_4_ and C_2_H_2_/C_2_H_4_. Grand canonical Monte Carlo (GCMC) simulations of binary mixtures containing 1:1 C_2_H_2_/C_2_H_4_, C_2_H_6_/C_2_H_4_, and CO_2_/C_2_H_4_ in Zn-atz-oba confirmed that these selectivities are greater than 1 at 298 K and 1 atm (Supplementary Table 5). These selectivities and relatively close adsorption enthalpies suggest that Zn-atz-oba might coadsorb the preferred sorbates C_2_H_2_, C_2_H_6_, and CO_2_ when subjected to a mixture feed.

### Mechanism of gas adsorption by GCMC simulations

**Fig. 3:**
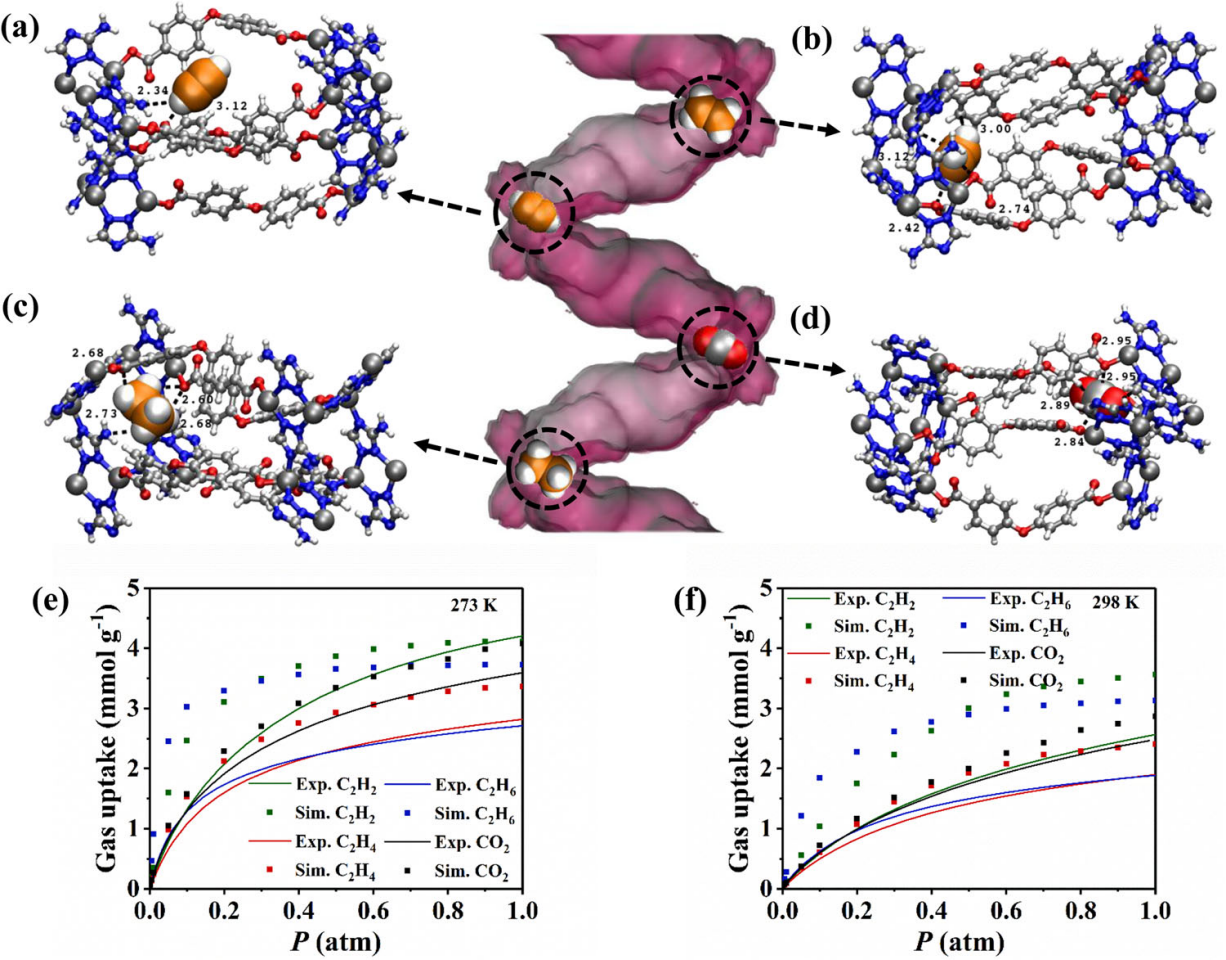
Molecular modeling guided determination of primary adsorption sites and adsorption isotherms in **Zn-atz-oba**. The primary adsorption sites of (**a**) C_2_H_2_, (**b**) C_2_H_4_, (**c**) C_2_H_6_, and (**d**) CO_2_ in Zn-atz-oba. Adsorbed gas molecules are presented in space-filling mode. color codes: C (Zn-atz-oba and CO_2_): gray; C (C2 gases): orange; H: white; O: red; N: blue; Zn: silver. Experimental (solid lines with circles) and simulated (squares) adsorption isotherms for C_2_H_2_ (green), C_2_H_4_ (red), C_2_H_6_ (blue), and CO_2_ (black) at (**e**) 273 K and (**f**) 298 K and pressures up to 1 atm in Zn-atz-oba.

GCMC simulations were conducted upon Zn-atz-oba at 273 and 298 K and afforded C_2_H_4_ and CO_2_ uptakes that are in good agreement with the corresponding experimental measurements at low pressure (≤0.2 atm), but are slightly higher than experimental values at higher pressures (Fig. 3). Moreover, it can be observed that the simulated uptakes for C_2_H_2_ and C_2_H_6_ significantly overestimate their corresponding experimental values for most of the state points considered. Since polarization contributes to less than 10% of the total energy for simulations of all four gases in Zn-atz-oba (see Supplementary Fig. 21), the overestimation of the theoretical uptakes compared with experiment might be attributable to the partial charges (Supplementary Data 1) and/or the repulsion/dispersion parameters (Supplementary Data 2) that were used for the MOF atoms (for crystallographic distances between MOF atoms see Supplementary Data 3; further details of the modeling study are provided in Supplementary Note 3: Modeling Study). Future work could exploit multiple MOF force field parameters for the simulations and investigate their effect upon the reliability of gas uptakes in this material. Although the simulated uptakes in Zn-atz-oba are inconsistent with experimental data, the simulations still properly predicted that this material exhibits the lowest uptake for C_2_H_4_ within the considered pressure range at 273 and 298 K. In addition, according to the simulations, Zn-atz-oba was expected to display higher uptake for C_2_H_6_ within the low-pressure region (<0.1 atm) compared to the other three gases. This is also consistent with the finding that this MOF exhibited the highest initial *Q*_st_ value toward C_2_H_6_ according to both experiment and simulation (Fig. 2d and Supplementary Table 4).

Molecular simulations revealed that the most favorable binding site for all adsorbates lies within the confined region enclosed by four atz linkers and three oba linkers (Fig. 3). In this region, C_2_H_2_ interacts with the –NH_2_ group of atz with N···H–C distances of 2.34 Å (Fig. 3a). C_2_H_4_, C_2_H_6_, and CO_2_ exhibit different orientations than C_2_H_2_, which allow them to make multiple contacts with the surrounding atoms of the framework. Most sorbent–sorbate interaction distances are longer for C_2_H_4_ versus C_2_H_6_ and CO_2_, indicating weaker interactions between C_2_H_4_ and the pores of Zn-atz-oba (Fig. 3b–d). The GCMC-calculated initial *Q*_st_ values were determined and exhibit the following trend: C_2_H_6_ > CO_2_ > C_2_H_2_ > C_2_H_4_ (see Supplementary Table 4). The greater *Q*_st_ value for C_2_H_6_ versus CO_2_ in Zn-atz-oba is supported by shorter interactions between C_2_H_6_ and Zn-atz-oba, perhaps because of the larger molecular dimensions of C_2_H_6_ and repulsive interactions between the negatively charged O atoms of CO_2_ and the surrounding electronegative N atoms of the atz linkers at the binding site. Further, the close N···H–C interactions exhibited by C_2_H_2_ are shorter than those of C_2_H_4_. Overall, the modeling studies support the experimental finding that C_2_H_4_ forms weaker interactions with Zn-atz-oba than the other three gases.

### Breakthrough experiments of Zn-atz-oba

**Fig. 4:**
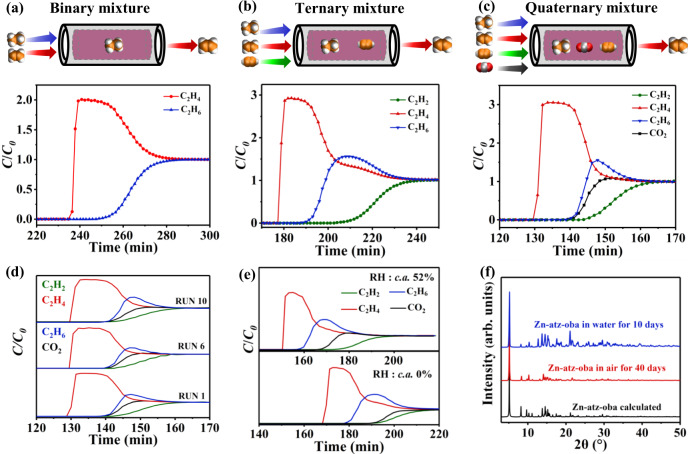
Dynamic breakthrough experiments and stability tests. (**a**)–(**c**) Experimental breakthrough curves at 298 K for C_2_H_4_/C_2_H_6_ (1:1) (**a**), C_2_H_2_/C_2_H_4_/C_2_H_6_ (1:1:1) (**b**), and C_2_H_2_/C_2_H_4_/C_2_H_6_/CO_2_ (1:1:1:1) (**c**) separations (equimolar mixtures; total gas pressure 100 kPa; total gas flow 1.4, 2.1 and 2.8 cm^3^ min^−1^, respectively) based on Zn-atz-oba (6.3 g) packed column (C: Outlet gas concentration, C_0_: Inlet gas concentration). (**d**) Dynamic breakthrough data obtained with Zn-atz-oba fixed-bed in 1^st^, 5^th^, and 10^th^ cycle when subjected to four-component C_2_H_2_/C_2_H_4_/C_2_H_6_/CO_2_ (1:1:1:1) mixture. **e** Four-component (1:1:1:1) breakthrough experiment in the presence of water vapor at 288 K and 100 kPa based on Zn-atz-oba (7.0 g) packed column (total gas flow of 2.8 cm^3^ min^−1^). (**f**) PXRD patterns of Zn-atz-oba after being subjected to air exposure and water immersion stability test conditions.

The gas separation performance was determined by dynamic column breakthrough experiments performed with Zn-atz-oba. In a typical breakthrough test at 298 K and ambient pressure, C_2_H_4_/C_2_H_6_, C_2_H_2_/C_2_H_4_/C_2_H_6_, and C_2_H_2_/C_2_H_4_/C_2_H_6_/CO_2_ mixtures (equimolar mixtures, total gas pressure = 100 kPa) were passed through a packed column and the effluent streams were monitored by gas chromatography. In accordance with the pure gas sorption results and the molecular simulations derived understanding of the binding sites, the Zn-atz-oba fixed-bed column eluted C_2_H_4_ with ultra-high purity in a single step from all three gas mixtures. As shown in Fig. 4a–c, C_2_H_4_ breaks through first at *ca*. 236, 177, and 130 min, respectively, and the impurities (C_2_H_6_, C_2_H_2_, and CO_2_) break through 14, 12, and 10 min later, respectively. Before breakthrough of C_2_H_6_, C_2_H_4_ of polymer grade purity (>99.95%) was collected at the outlet, revealing that Zn-atz-oba achieved one-step purification of C_2_H_4_ by the coadsorption of C_2_H_2_, C_2_H_6_, and CO_2_ from C_2_H_2_/C_2_H_4_/C_2_H_6_/CO_2_ (>99.95% C_2_H_4_ productivity, 0.106 mmol g^−1^). To explore the recycling performance of Zn-atz-oba, ten cycles of four-component breakthrough experiments were conducted. There was no loss of C_2_H_4_ retention time (Fig. 4d). In industrial C2 hydrocarbon gas streams, C_2_H_2_ only constitutes ~1% of the total flow^50^. We also tested a C_2_H_2_/C_2_H_4_/C_2_H_6_/CO_2_ (1/33/33/33) mixture under dynamic breakthrough using 7.4 g Zn-atz-oba packed in a fixed-bed. As shown in Fig. S25, polymer-grade ethylene was harvested from 1/33/33/33 gas mixture, followed by C_2_H_6_, CO_2,_ and C_2_H_2_. As a typical contaminant in downstream feedstocks, water vapor is present in the industrially produced C2-CO_2_ stream^51^, so breakthrough experiments in the presence of water vapor were conducted. As revealed by Fig. 4e, fractional pressure reduction of C_2_H_2_, C_2_H_4_, C_2_H_6_, and CO_2_ in the presence of water vapor reduced the adsorption capacity of Zn-atz-oba. This is reflected in a reduced C_2_H_4_ breakthrough time, from *c.a*. 170 min to *c.a*. 150 min. 4-component C_2_H_2_/C_2_H_4_/C_2_H_6_/CO_2_ (1:1:1:1) breakthrough experiments in the presence of water vapor (relative humidity 52%) also resulted in a reduced retention time but a consistent gas outflow sequence. Nevertheless, C_2_H_4_ with effluent purity >99.95% was collected at the column outlet. We attribute the shorter retention time under 52% humidity to the presence of water vapor in the breakthrough pipeline and competing sorption of water with the other four gases. After ambient air exposure for 30 days or water immersion for 10 days, PXRD patterns (Fig. 4f) and N_2_ isotherms at 77 K (including pore distribution, Supplementary Figs. 26 and 27) of regenerated Zn-atz-oba indicated that it is stable to both humid air and liquid water. Temperature-programmed desorption of the fully adsorbed column was conducted after achieving full saturation and revealed that the Zn-atz-oba bed could be regenerated within 50 min at 333 K under He flow (30 cm^3^ min^−1^). Even under ambient conditions (298 K and 100 kPa), regeneration was achieved within 140 min under He flow of 20 cm^3^ min^−1^ (Supplementary Fig. 23), confirming that there is indeed a relatively low energy footprint for Zn-atz-oba regeneration.

## Discussion

**Fig. 5:**
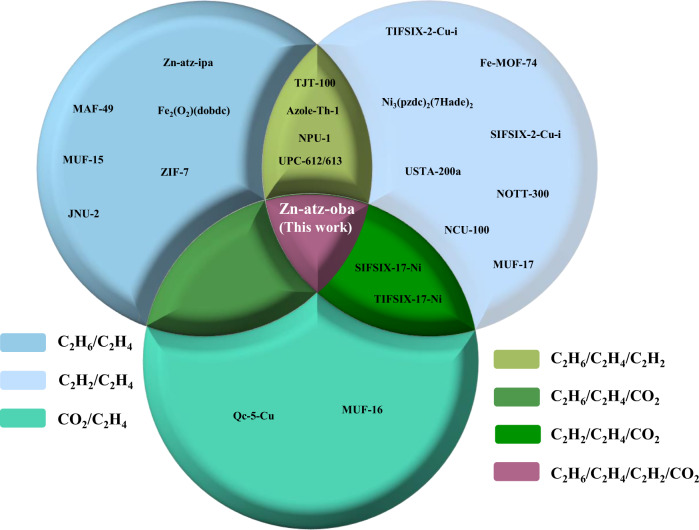
Physisorbents studied for C_2_H_4_ purification. Comparison of physisorbents that can purify C_2_H_4_ from binary (sky blue)^15–20, 22–30, 32, 46^, ternary (olive green)^35, 36, 39–41^ and quaternary mixtures (lilac, this work) under ambient conditions.

If one compares the performances of previously reported physisorbents in the context of ethylene purification from binary mixtures^15–20,22–30,32,46^ (C_2_H_2_/C_2_H_4_, C_2_H_6_/C_2_H_4_, and C_2_H_4_/CO_2_) and ternary mixtures^35,36,39–41^ (C_2_H_2_/C_2_H_4_/C_2_H_6_, C_2_H_2_/C_2_H_4_/CO_2_, and C_2_H_6_/C_2_H_4_/CO_2_), it is evident that Zn-atz-oba represents a benchmark in terms of its performance parameters (Fig. 5). Specifically, Zn-atz-oba coadsorbs three gases, C_2_H_2_, C_2_H_6_, and CO_2_, to produce polymer-grade (>99.95%) C_2_H_4_ in just one step. We attribute this performance to the unusual pore environment in Zn-atz-oba, which enables roughly equal affinity towards C_2_H_2_, C_2_H_6_, and CO_2_ over C_2_H_4_. Future studies will focus upon crystal engineering of second-generation variants to further improve the purification performances of other gas mixtures of industrial relevance.

## Methods

### General

All reagents were obtained from vendors and used as received without further purification. Powder X-ray diffraction (PXRD) data were collected with a Rigaku-Miniflex-600 diffractometer at a scanning rate of 5° min^−1^ using Cu K_α_ radiation. Thermogravimetric analysis (TGA) data were obtained using Mettler TG DSC 3+ Thermogravimetric Analyzer. In TGA measurements, the sample was heated from 298 K to 1073 K under Ar atmosphere at a heating rate of 10 K min^−1^. The morphology was examined using scanning electron microscopy (FESEM, Verios G4, FEI).

### Synthesis of Zn-atz-oba

A mixture of Zn(NO_3_)_2_·6H_2_O (10 mmol, 2.98 g), H_2_oba (5 mmol, 1.29 g), Hatz (10 mmol, 0.84 g), DMF (40 mL), MeOH (40 mL), and H_2_O (20 mL) was sealed in a 250 ml borosilicate bottle and sonicated for five minutes. The reaction mixture was then solvothermally treated at 403 K for 3 days before naturally cooling it to room temperature. The as-synthesized sample Zn-atz-oba was washed with fresh DMF, MeOH, and H_2_O, and exchanged with MeOH three times daily for three days and then activated at 353 K in vacuum for 12 h.

### Single-component gas sorption experiments

A Micromeritics 3Flex was used for recording all gas sorption isotherms, N_2_ (77 K), C_2_H_2_, C_2_H_4_, C_2_H_6_, and CO_2_ (each, 273 and 298 K). For N_2_ adsorption isotherms, the temperature was controlled at 77 K using a Dewar containing 4 L liquid N_2_. Precise control of 273 and 298 K temperatures was implemented by a dc-2006 from Ningbo Scientz Biotechnology, which contained a cyclic control system of ethylene glycol and water mixture (v/v = 1:1). Zn-atz-oba was degassed at 298 K under high vacuum for 4 h to regenerate in between consecutive isotherm measurements.

### Dynamic gas breakthrough experiments

Breakthrough curves were recorded by an in-house custom-built rig (Supplementary Fig. 22). Equimolar C_2_H_6_/C_2_H_4_ (1:1), C_2_H_2_/C_2_H_4_/C_2_H_6_ (1:1:1) and C_2_H_2_/C_2_H_4_/C_2_H_6_/CO_2_ (1:1:1:1) gas mixtures (total gas pressure and flow: 100 kPa and 1.4, 2.1, and 2.8 cm^3^ min^−1^, respectively) were subjected through the Zn-atz-oba packed column (6.3 g) at 298 K, and the outlet gas concentrations and composition were monitored by a gas chromatography analyzer (Carrier gas: He, TCD-Thermal Conductivity Detector, detection limit 0.1 ppm). During gas breakthrough cycling tests, Zn-atz-oba packed in the column was regenerated under He flow of 30 cm^3^ min^−1^ at 333 K for 2 h, after each breakthrough experiment.

## Supplementary information

Supplementary Information

Description of Additional Supplementary Files

Supplementary Data 1

Supplementary Data 2

Supplementary Data 3

## Data Availability

The sorption data; molecular simulations and breakthrough data generated in this study are provided in the Supplementary Information/Source Data file^52^.

## Code availability

The codes used to analyse all the data generated in this study are provided in the Supplementary Information (Supplementary Note 1: Calculations, Supplementary Note 2: Adsorption enthalpy calculation and Supplementary Note 3: Modeling Study) and/or the Source Data file^52^.
