# Supplementary material for: One-step ethylene production from a four-component gas mixture by a single physisorbent
Source: Nat Commun. 2021 Nov 11;12:6507. doi: 10.1038/s41467-021-26473-8 (PMC8586343; doi:10.1038/s41467-021-26473-8)
Supplement: Supplementary file 1 — Supplementary Information [file 41467_2021_26473_MOESM1_ESM.pdf]

Supplementary Information

One-step Ethylene Production from a Four-component Gas Mixture by a Single Physisorbent

Jian-Wei Cao,[1,†] Soumya Mukherjee,[2,3,†] Tony Pham,[4] Yu Wang,[1] Teng Wang,[1] Tao Zhang,[1] Xue Jiang,[1] Hui-Juan Tang,[1] Katherine A. Forrest,[4] Brian Space,[4,5] Michael J. Zaworotko,[2,*] and Kai-Jie Chen[1,*]

[1]Key Laboratory of Special Functional and Smart Polymer Materials of Ministry of Industry and Information Technology, Xi'an Key Laboratory of Functional Organic Porous Materials, School of Chemistry and Chemical Engineering, Northwestern Polytechnical University, Xi'an, Shaanxi 710072, P.R. China.

[2]Bernal Institute, Department of Chemical Sciences; University of Limerick; Limerick V94 T9PX, Republic of Ireland.

[3]Department of Chemistry, Technical University of Munich, Lichtenbergstraße 4, 85748 Garching b. München, Germany.

[4]Department of Chemistry, University of South Florida, 4202 East Fowler Avenue, CHE205, Tampa, Florida 33620-5250, USA.

[5]Department of Chemistry, North Carolina State University, USA.

***Corresponding authors**: xtal@ul.ie; ckjiscon@nwpu.edu.cn

[†]J.W.C. and S.M. have contributed equally.

Supplementary Note 1: Calculations

Calculation of surface area from single crystal

$$S(m^2g^{-1}) = \frac{Surface\ area\ per\ cell\ (\text{Å}^2)}{Density\ (g\ cm^{-3}) \times Cell\ volume\ (\text{Å}^3)} \times 10^4 \qquad \text{(Supplementary Equation 1)}$$

Calculation of pore volume from single crystal

$$V_p = \frac{Cell\ free\ volume\ (\text{Å}^3)}{Density\ (g\ cm^{-3}) \times Cell\ volume\ (\text{Å}^3)} \qquad \text{(Supplementary Equation 2)}$$

Calculation of surface area from nitrogen isotherm

Langmuir model

$$\frac{P}{V} = \frac{1}{V_m b} + \frac{P}{V_m} \qquad \text{(Supplementary Equation 3)}$$

P is the gas pressure in kPa, V_m is saturated adsorption capacity of the monolayer in cm^3. *V* is the adsorption capacity in cm^3 when the gas pressure is *P*. *b* is a constant associated with the adsorbent.

BET model

$$\frac{P}{V(P_0 - P)} = \frac{1}{V_m C} + \frac{(C-1)P}{V_m C P_0} \qquad \text{(Supplementary Equation 4)}$$

P is the gas pressure in kPa, P_0 is a saturated vapor pressure for nitrogen at 77 K, V_m is the saturated adsorption capacity of the monolayer in cm^3. *V* is the adsorption capacity in cm^3 when the gas pressure is *P*, *C* is a constant associated with the adsorbent.

Calculation of surface area

$$S = \frac{V_m N_A \delta}{m \times 22400} \qquad \text{(Supplementary Equation 5)}$$

N_A is Avogadro constant (6.023×10^{23} mol^{-1}). V_m is saturated adsorption capacity of the monolayer in cm^3. *m* is mass of sample in g, δ is the section area of nitrogen molecules in m^2.

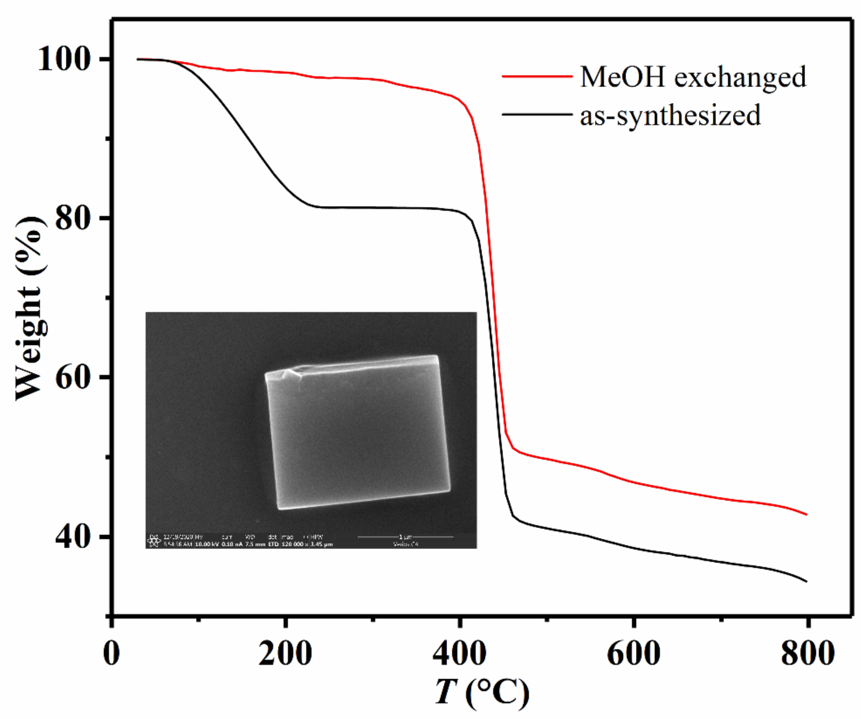

Supplementary Figure 1. TG analysis of Zn-atz-oba. As-synthesized and the MeOH soaked samples under Ar atmosphere at a heating rate of 10 ºC per minute. The inset is the FESEM image of as-synthesised Zn-atz-oba**.**

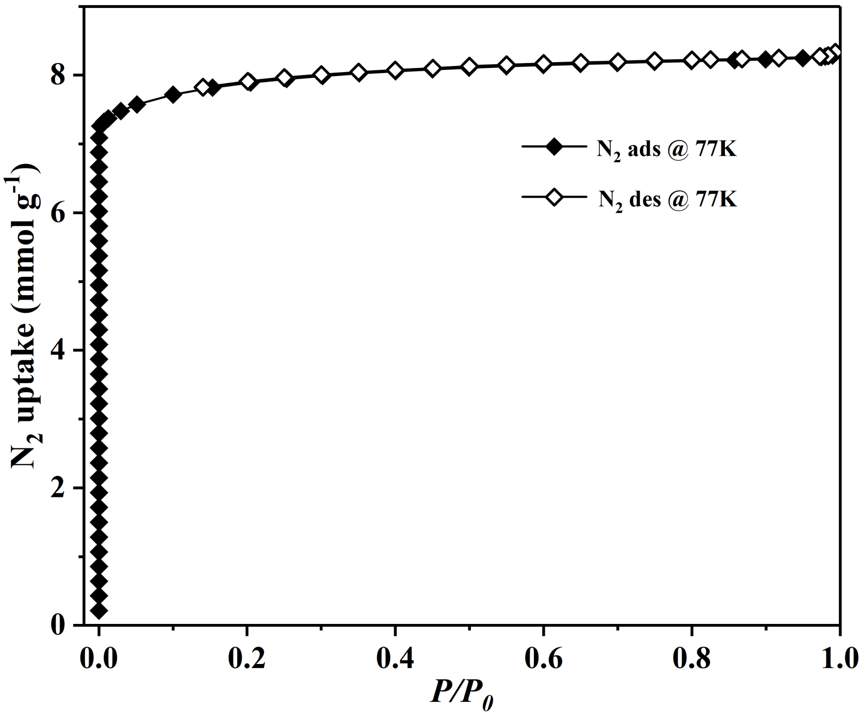

Supplementary Figure 2. N_2 sorption isotherm of Zn-atz-oba at 77 K.

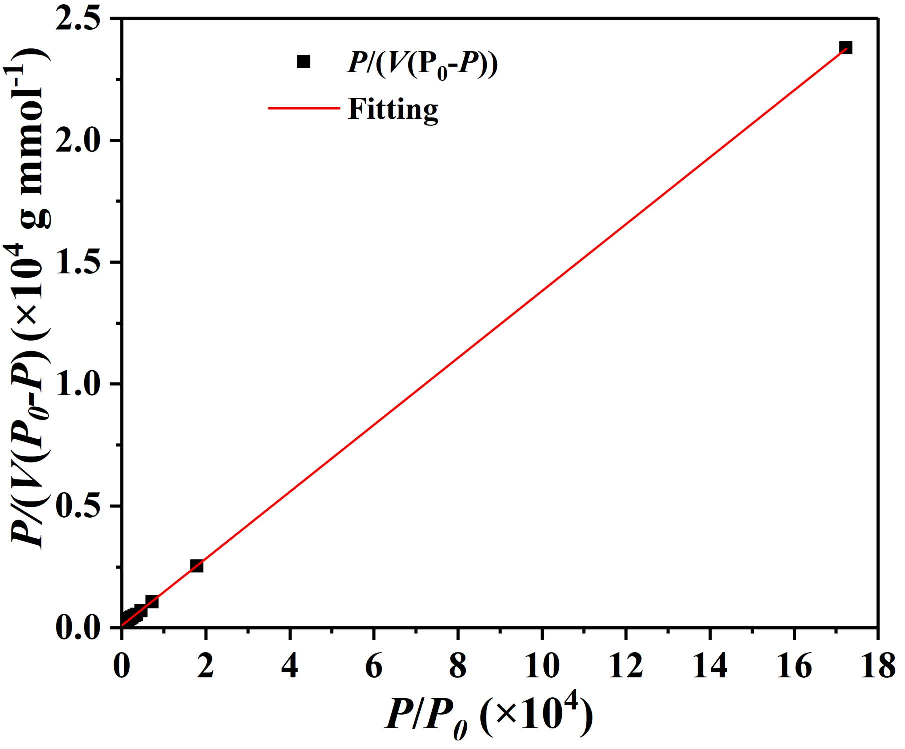

Supplementary Figure 3. BET fitting from N_2 isotherm of Zn-atz-oba at 77 K.

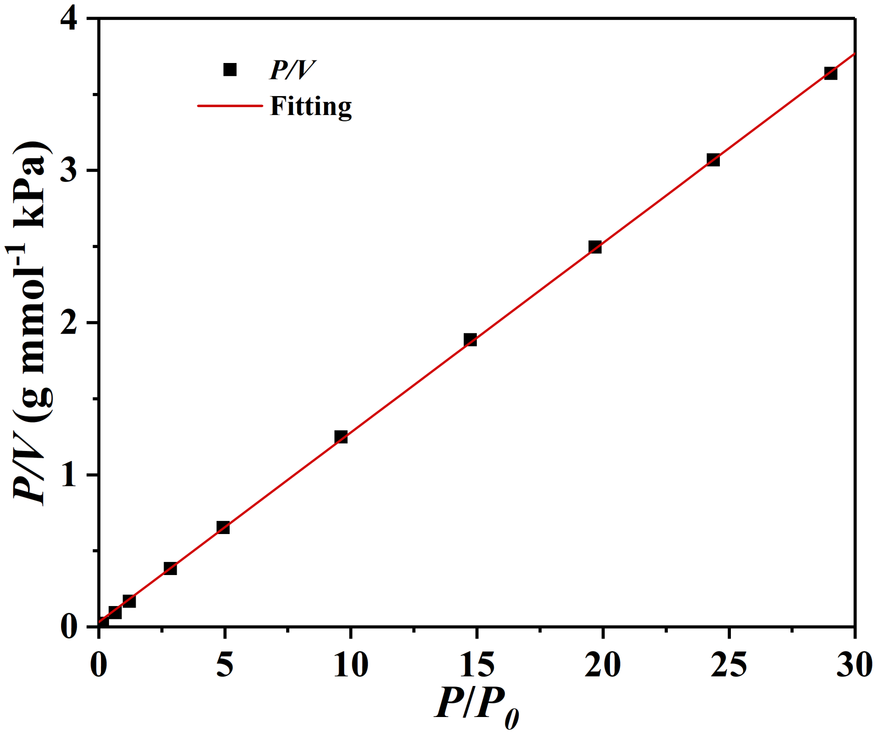

Supplementary Figure 4. Langmuir fitting from N_2 isotherm of Zn-atz-oba at 77 K.

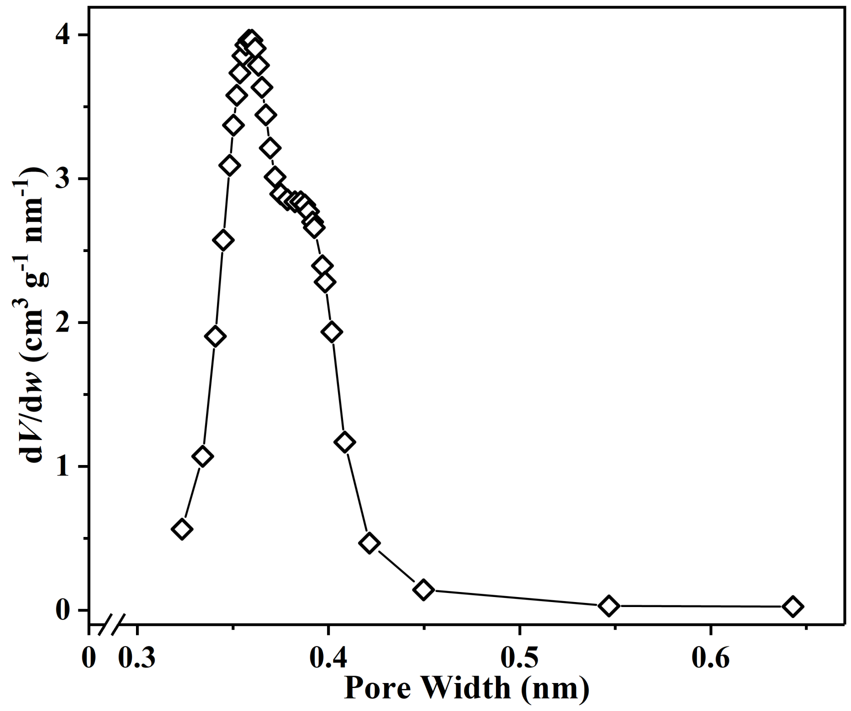

Supplementary Figure 5. Pore size distribution profile of Zn-atz-oba calculated by the H-K model (pore geometry: silt).

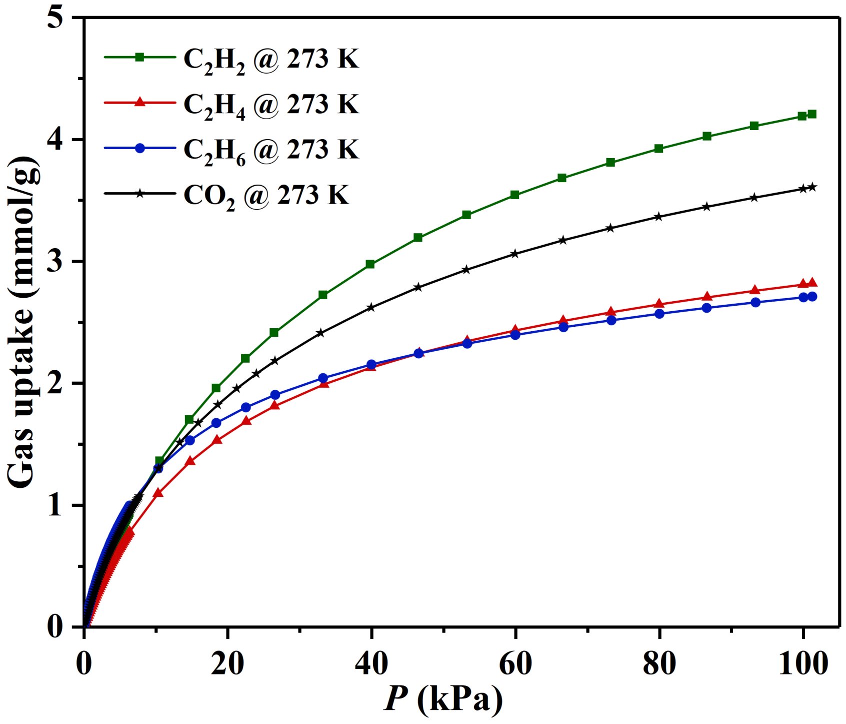

Supplementary Figure 6. For Zn-atz-oba, C_2H_2, C_2H_4, C_2H_6 and CO_2 adsorption isotherms recorded at 273 K.

Supplementary Note 2: Adsorption enthalpy calculation

Adsorption enthalpy of adsorption was calculated by virial equation using the isotherms recorded at 273 and 298 K.

$$ln\,P = ln\,N + \left(\sum_{i=0}^{m} a_i N^i\right)/T + \sum_{i=0}^{n} \binom{n}{k} b_i N^i \qquad \text{(Supplementary Equation 6)}$$

$$Q_{st} = -R \sum_{i=0}^{m} a_i N^i \qquad \text{(Supplementary Equation 7)}$$

P is the pressure described in Pa, *N* is the adsorbed amount in mmol/g, *T* is the temperature in K, a_i and b_i are virial coefficients, and *m* and *n* are the numbers of coefficients used to describe the isotherms. Q_{st} is the coverage-dependent enthalpy of adsorption and *R* is the universal gas constant.

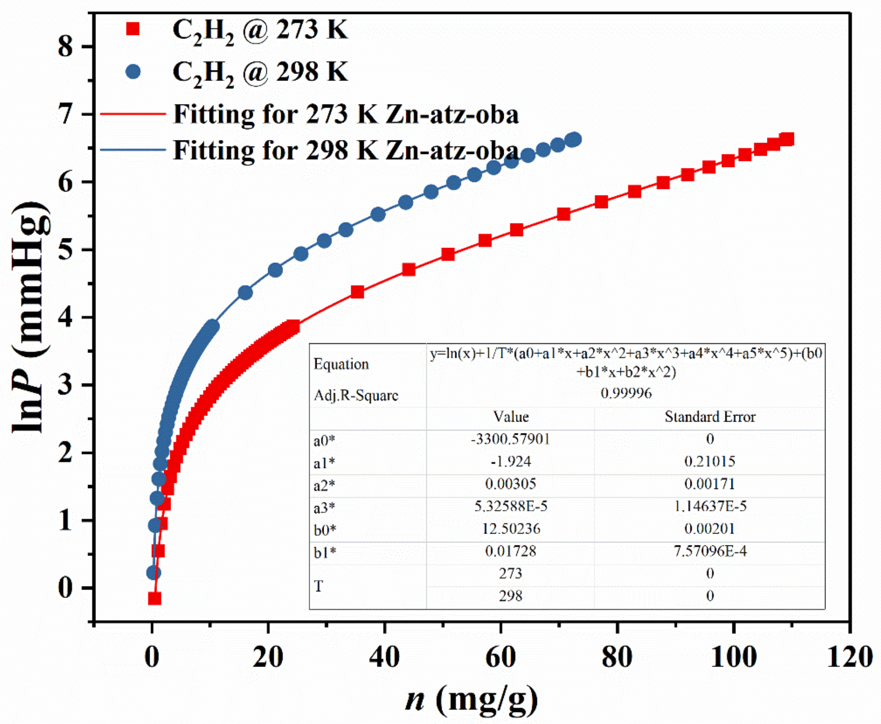

Supplementary Figure 7. Virial fitting of C_2H_2 adsorption data for Zn-atz-oba.

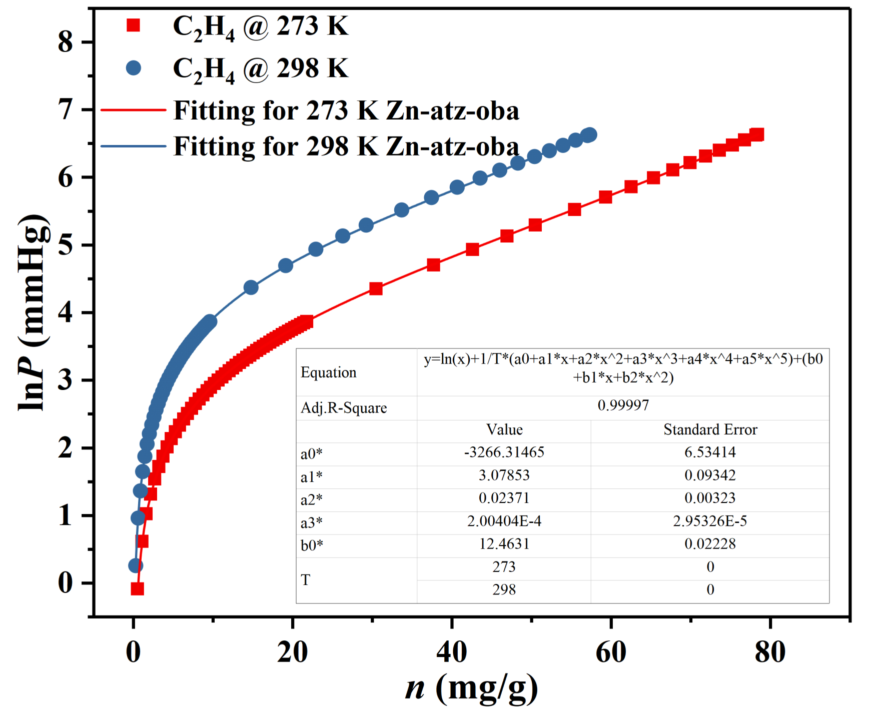

Supplementary Figure 8. Virial fitting of C_2H_4 adsorption data for Zn-atz-oba.

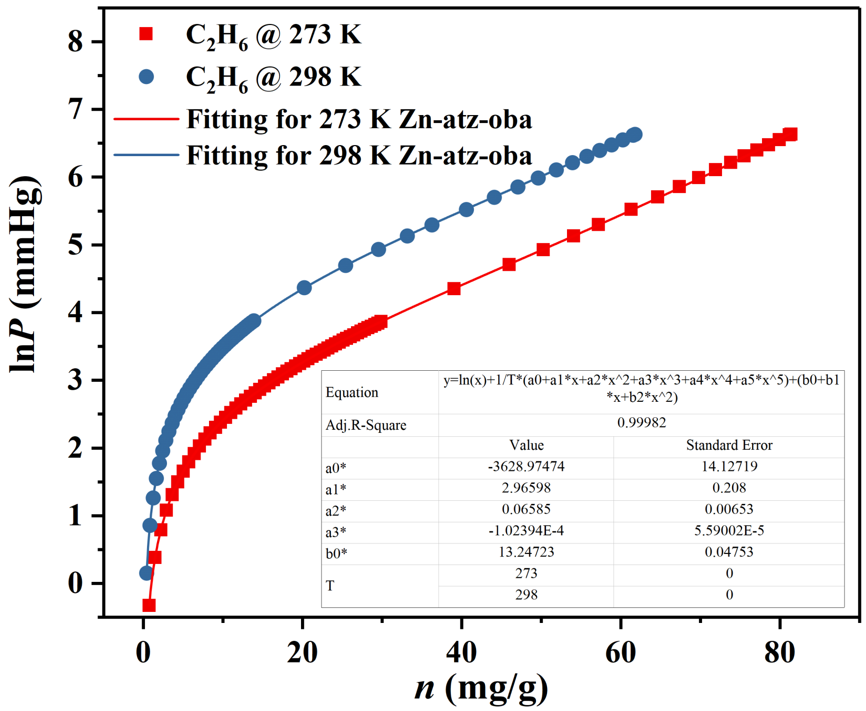

Supplementary Figure 9. Virial fitting of C_2H_6 adsorption data for Zn-atz-oba.

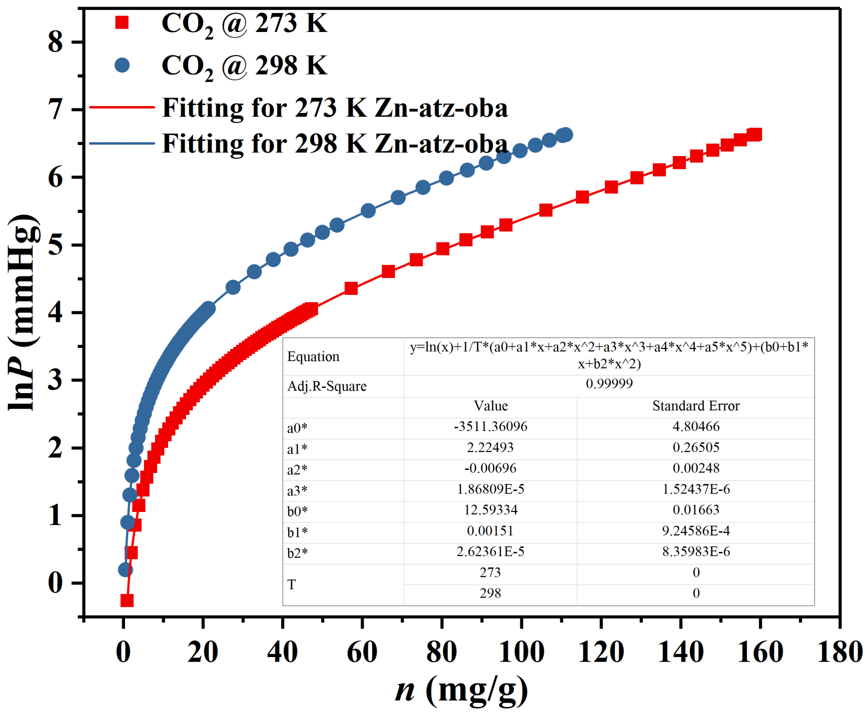

Supplementary Figure 10. Virial fitting of CO_2 adsorption data for Zn-atz-oba.

Langmuir-Freundlich fit

Adsorption isotherms for C_2H_6, C_2H_4 and C_2H_2 in **Zn-atz-oba** were fitted to the single-site Langmuir-Freundlich model.

$$q = Q_{sat} \frac{b_A p^v}{1+b_A p^v} \quad \text{(Supplementary Equation 8)}$$

b_A is Langmuir-Freundlich constant for species i at adsorption site A (Pa^{-v}). Q_{sat} is saturation loading ($mol\ kg^{-1}$). q_i is molar loading of species i ($mol\ kg^{-1}$). P is the total pressure (in kPa) of the bulk gas at equilibrium with the adsorbed phase, v is Freundlich exponent (dimensionless).

IAST selectivity calculation

Adsorption selectivity of C_2H_2/C_2H_4, C_2H_6/C_2H_4, and CO_2/C_2H_4 mixed gases was predicted from single component adsorption isotherms using Ideal Adsorbed Solution Theory (IAST)[1].

$$S_{AB} = \frac{x_A/x_B}{y_A/y_B} \quad \text{(Supplementary Equation 9)}$$

Where S_{AB} is the adsorption selectivity of component A relative to B. X_A and X_B are the molar fractions of components A and B in the adsorption phase, respectively. Y_A and Y_B are molar fractions of components A and B in the gas phase, respectively.

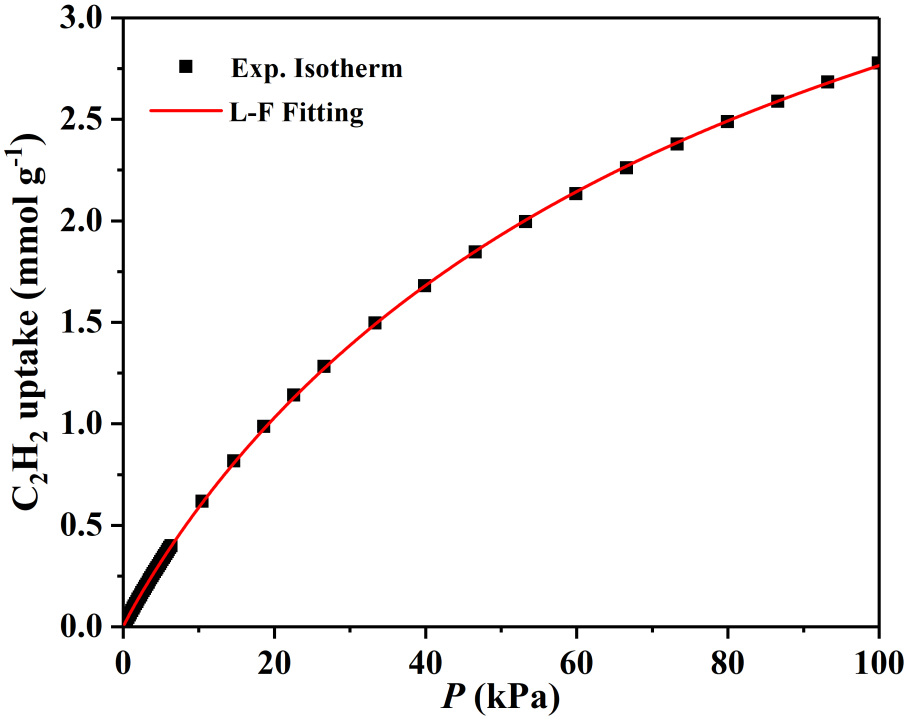

Supplementary Figure 11. Langmuir-Freundlich fitting of C_2H_2 adsorption isotherm at 298 K for Zn-atz-oba.

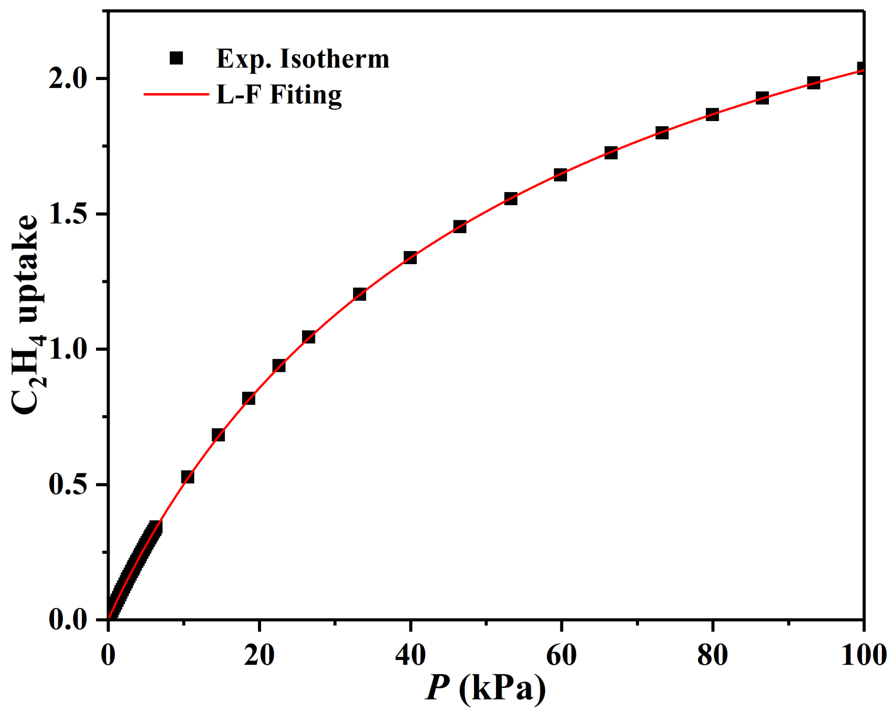

Supplementary Figure 12. Langmuir-Freundlich fitting of C_2H_4 adsorption isotherm at 298 K for Zn-atz-oba.

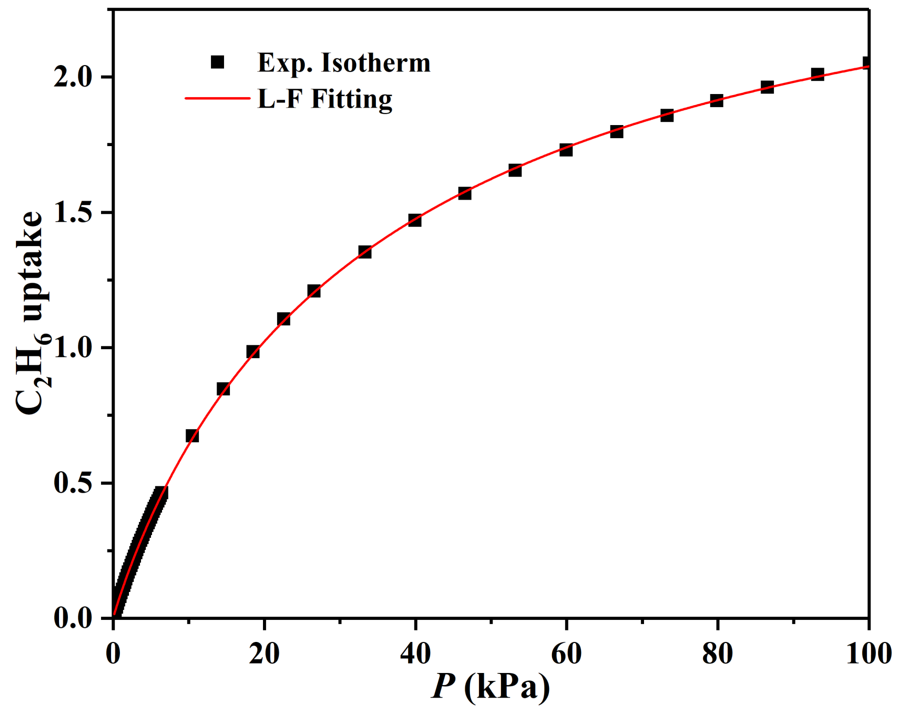

Supplementary Figure 13. Langmuir-Freundlich fitting of C_2H_6 adsorption isotherm at 298 K for Zn-atz-oba.

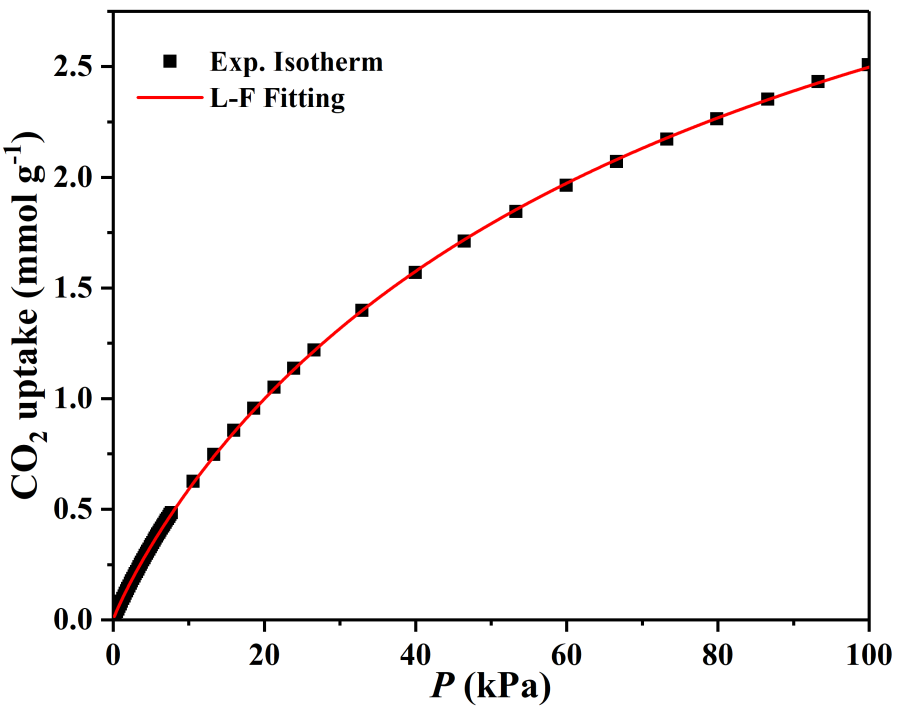

Supplementary Figure 14. Langmuir-Freundlich fitting of C_2H_6 adsorption isotherm at 298 K for Zn-atz-oba.

Supplementary Table 1. Langmuir-Freundlich fitting parameters of C_2H_2, C_2H_4, C_2H_6 and CO_2 adsorption at 298K for **Zn-atz-oba**.

**Adsorbates**	Q_{sat}	b_A	**V**
**C_2H_2**	5.09948	0.01432	0.95884
**C_2H_4**	3.14541	0.01966	0.98356
**C_2H_6**	2.81133	0.03313	0.95091
**CO_2**	4.49627	0.01825	0.91782

Supplementary Table 2. Sorption data summary of **Zn-atz-oba**.

<table>
<tr><th></th><th colspan="2">C_2H_6</th><th colspan="2">C_2H_4</th><th colspan="2">C_2H_2</th><th colspan="2">CO_2</th></tr>
<tr><td>273 K uptake[a] (mmol·g^{-1})</td><td colspan="2">1.27/2.28/2.70</td><td colspan="2">1.06/2.29/2.81</td><td colspan="2">1.31/3.30/4.19</td><td colspan="2">1.27/2.86/3.59</td></tr>
<tr><td>298 K uptake[b] (mmol·g^{-1})</td><td colspan="2">0.64/1.61/2.05</td><td colspan="2">0.50/1.50/2.03</td><td colspan="2">0.59/1.92/2.77</td><td colspan="2">0.60/1.78/2.50</td></tr>
<tr><td>Low loading Q_{st} (kJ·mol^{-1})</td><td colspan="2">30.05</td><td colspan="2">27.07</td><td colspan="2">27.49</td><td colspan="2">29.08</td></tr>
<tr><td rowspan="2">IAST selectivity[c]</td><td colspan="3">C_2H_6/C_2H_4</td><td colspan="2">C_2H_2/C_2H_4</td><td colspan="3">CO_2/C_2H_4</td></tr>
<tr><td colspan="3">1.27</td><td colspan="2">1.43</td><td colspan="3">1.33</td></tr>
</table>

a: Uptake at 0.1/0.5/1.0 bar and 273 K;

b: Uptake at 0.1/0.5/1.0 bar and 298 K;

c: IAST selectivity of 1:1 gas mixture at 298 K and 1.0 bar.

Supplementary Table 3. Table of IAST selectivity of benchmark dual-component (C_2H_2/C_2H_4, C_2H_6/C_2H_4), three-component ($C_2H_2/C_2H_4/C_2H_6$) separation adsorbent and this work ($C_2H_2/C_2H_4/C_2H_6/CO_2$ four-component).

MOF	IAST selectivity at 1 bar			References
	C_2H_6/C_2H_4	C_2H_2/C_2H_4	CO_2/C_2H_4	
**UTSA-300a**	--	~10^4 [a]	--	2
**TIFSIX-2-Cu-i**	--	667.0[b]	--	3
**SIFSIX-2-Cu-i**	--	41.01[b]	--	4
**NOTT-300**	--	2.3[b]	--	5
**Fe-MOF-74**	--	2.1[b]	--	6
**UTSA-30**	3.8[b]	--	--	7
**MAF-49**	2.7[b]	--	--	8
**Zn-atz-ipa**	1.7[b]	--	--	9
**JNU-2**	1.6[b]	--	--	10
**MUF-16**	--	--	600[b]	11
**Qc-5-Cu**	--	--	39.95[a]	12
**TIFSIX-17-Ni**	--	667.0[b]	148.4[b]	13
**SIFSIX-17-Ni**	--	503.5[b]	98.1[b]	
**TJT-100**	1.2[b]	1.8[b]	--	14
**Azole-Th-1**	1.46[b]	1.09[b]	--	15
**NPU-1**	1.32[b]	1.4[b]	--	16
**UPC-612**	1.4[b]	1.07[b]	--	17
**UPC-613**	1.5[b]	1.4[b]	--	
**Zn-atz-oba**	1.27[b]	1.43[b]	1.33[b]	**This work**

[a]IAST selectivity for 1/99 gas mixture. [b]IAST selectivity for 1/1 gas mixture.

Supplementary Note 3: Modeling Study

The single X-ray crystallographic structure of Zn-atz-oba published in reference 47 (CCDC 944515) was used for the parametrizations and simulations.

All atoms of Zn-atz-oba were treated with Lennard-Jones (LJ) 12–6 parameters (ε and σ),[19] point partial charges, and static point polarizabilities to model repulsion/dispersion, stationary electrostatic, and many-body polarization interactions, respectively. The LJ parameters for all aromatic C and H atoms were taken from the Optimized Potentials For Liquid Simulations – All Atom (OPLS-AA) force field,[20] while such parameters for all other atoms were taken from the Universal Force Field (UFF).[21] Examination of the crystal structure of Zn-atz-oba revealed 47 atoms in chemically distinct environments (Supplementary Figure 15). The partial charges for each unique atom were determined through electronic structure calculations on different gas phase fragments that were extracted from the crystal structure of the MOF. Six fragments were considered for the calculations in this work and they are shown in Supplementary Figure 16.

For these calculations, all C, H, N, and O atoms were treated with the 6-31G* basis set,[22, 23] while the LANL2DZ ECP basis set[24-26] was used for the Zn^{2+} ions. The NWChem *ab initio* software[27] was used to calculate the electrostatic potential surface for each fragment and the partial charges were subsequently fitted onto the atomic positions of the fragments using the CHELPG method.[28, 29] The partial charges for all chemically distinct atoms were averaged between the fragments. It can be observed that excellent agreement was obtained for the partial charges for the unique atoms between the fragments, with standard deviations of no greater than 0.1 e^{-} (see Supplementary Data 1). When considering the average partial charges for the chemically distinct atoms between the fragments and the number of each type of atom within the unit cell, the calculated total charge of the system was not neutral. Since the magnitude of the total negative charge outweighed the magnitude of the total positive charge in this case, all unique atoms with negative charges were multiplied by a factor (the absolute value of the ratio of total positive charge to total negative charge) to bring the magnitude of the total negative charge to be equivalent with that for the total positive charge. The resulting partial charges for each chemically distinct atom in Zn-atz-oba after the adjustment can be found in Supplementary Data 2. These partial charges were used for the simulations in this work to calculate stationary electrostatic interactions.

Although the partial charges for the MOF atoms can be determined through other methods that involve periodic fitting of the entire unit cell,[30–32] utilizing partial charges that have been obtained

through calculations on fragments has shown to generate simulated results that are in good agreement with experimental measurements in certain cases.[33,34] Notably, the set of partial charges employed herein for Zn-atz-oba led to simulated results that are in reasonable agreement with experimental measurements and reproduced an important experimental finding.

In order to model explicit many-body polarization interactions, static point polarizabilities were assigned to the nuclear center of all atoms of Zn-atz-oba. The exponential damping-type polarizability values for all C, H, N, and O atoms were taken from a carefully parametrized set provided by the work of van Duijnen and Swart.[35] The polarizability parameter for Zn^{2+} was calculated in previous work[36,37] and used herein. The simulation parameters used here for Zn-atz-oba are presented in Supplementary Data 2. The crystallographic distances between various unique atoms for the MOF are provided in Supplementary Data 3.

Classical Monte Carlo (MC) simulations of C_2H_2, C_2H_4, C_2H_6, and CO_2 adsorption were performed in Zn-atz-oba within a rigid 2 × 2 × 2 supercell of the MOF. A spherical cut-off distance corresponding to half the shortest supercell dimension length was used for the simulations. C_2H_2, C_2H_4, C_2H_6, and CO_2 were modeled using polarizable potentials of the respective adsorbates that were developed previously.[38,39] The total potential energy of the MOF–adsorbate system was calculated through the sum of the repulsion/dispersion, stationary electrostatic, and many-body polarization energies. These were calculated using the Lennard-Jones 12–6 potential,[3] partial charges with Ewald summation,[40,41] and a Thole-Applequist type model,[42-45] respectively. All MC simulations were performed using the Massively Parallel Monte Carlo (MPMC) code.[46,47]

In order to identify the global energy minimum for C_2H_2, C_2H_4, C_2H_6, and CO_2 in Zn-atz-oba, simulated annealing (SA) calculations[48] were performed for a single molecule of each adsorbate through a canonical Monte Carlo (*NVT*) process in the considered supercell of the MOF. SA calculations for each adsorbate utilized an initial temperature of 500 K, and this temperature was scaled by a factor of 0.99999 after every 10^3 MC steps. The simulations continued until 10^6 MC steps were reached; at this point, the temperature of the system is below 15 K and the adsorbate is already localized in its energy minimum position in the MOF.

Simulated adsorption isotherms for C_2H_2, C_2H_4, C_2H_6, and CO_2 in $[Zn_2(atz)_2(oba)]$ at 273 and 298 K and pressures up to 1 atm were generated using grand canonical Monte Carlo (GCMC) methods.[49] The theoretical Q_{st} value at zero-coverage (Q^0_{st}) for all four adsorbates in Zn-atz-oba were also estimated through performing GCMC simulations at 273 K and 0.001 atm. This quantity was calculated using a statistical mechanical expression based on fluctuations in the particle number and total potential energy of the system.[50] GCMC simulations were also carried out at 195 K and 1.0 atm in order to obtain the modeled structure at saturation for each adsorbate. For all state points considered, the simulations consisted of 2.5 × 10^6 MC steps to guarantee equilibration, followed by an additional 2.5 × 10^6 steps to ensure

reasonable ensemble averages for the particle number and Q^0_{st}. Once the average particle number was calculated, it was converted to a value that is equivalent with an experimental quantity for gas uptake for each state point considered. A comparison of the experimental and simulated adsorption isotherms for C_2H_2, C_2H_4, C_2H_6, and CO_2 in Zn-atz-oba at 273 and 298 K are shown in Figure 3. The calculated Q^0_{st} values for all four adsorbates in Zn-atz-oba are listed in Supplementary Table 4 and are in good agreement with the corresponding experimental values. The relative trend in the theoretical Q^0_{st} values is also consistent with experiment. According to the simulations, saturation of C_2H_2, C_2H_4, C_2H_6, and CO_2 in Zn-atz-oba is achieved at 13, 11, 10, and 15 molecules per unit cell, respectively. The modeled 2 × 2 × 2 supercell of Zn-atz-oba containing the saturated loading amount for C_2H_2, C_2H_4, C_2H_6, and CO_2 are shown in Supplementary Figure 17, 18, 29, and 20, respectively.

GCMC simulations of binary mixtures containing 50:50 compositions of C_2H_2/C_2H_4, C_2H_6/C_2H_4, and CO_2/C_2H_4 were also performed in Zn-atz-oba at 298 K and 1 atm. These simulations were performed in the ideal gas limit where the fugacities were equal to the partial pressures. The selectivity of one adsorbate molecule relative to another was calculated by the following expression:

$$S = \frac{x_i \, y_j}{x_j \, y_i} \qquad \text{(Supplementary Equation 10)}$$

Supplementary Equation 10 refers where x_i and x_j are the mole fractions of components i and j, respectively, in the adsorbed phase and y_i and y_j are the mole fractions of components i and j, respectively, in the bulk phase. The calculated 50:50 mixture selectivities for C_2H_2/C_2H_4, C_2H_6/C_2H_4, and CO_2/C_2H_4 in Zn-atz-oba at the state point considered are presented in Supplementary Table 5.

Supplementary Figure 21 shows the averaged percent contribution of the three energetic terms (repulsion/dispersion, permanent electrostatics, and polarization) per adsorbate molecule for C_2H_2, C_2H_4, C_2H_6, and CO_2 adsorption in Zn-atz-oba at 298 K and pressures up to 1 atm. It can be observed that repulsion/dispersion interactions contribute to roughly 67–71% of the total energy for C_2H_2 adsorption in Zn-atz-oba within the considered pressure range (Supplementary Figure 21(a)). The electrostatic contribution for this adsorbate is about 20–25% within the same pressure range. For CO_2 adsorption, repulsion/dispersion and electrostatic interactions contribute to approximately 65–67% and 24–26% of the total energy, respectively (Supplementary Figure 21(d)). Overall, it appears that electrostatic interactions represent nearly 25% of the total energy for C_2H_2 and CO_2

adsorption in Zn-atz-oba, suggesting that this energetic term signifies an important contribution to the adsorption mechanism for these gases. C_2H_6 adsorption in Zn-atz-oba is dominated by repulsion/dispersion interactions as the percent contribution for this energetic term is over 95% for each state point considered (Supplementary Figure 21(c)). This could be due to the fact that C_2H_6 contains multiple atoms that can interact simultaneously with the surrounding framework atoms at the primary binding site through strong repulsion/dispersion interactions. For C_2H_4 adsorption, the contribution from repulsion/dispersion interactions is 86–88%, while that for electrostatic interactions is 7–8% (Supplementary Figure 21(b)). Zn-atz-oba displays the weakest interaction toward C_2H_4 possibly due to low contributions from electrostatic interactions and repulsion/dispersion percentages that are not as high as those for C_2H_6. Finally, it can be observed that polarization interactions contribute negligibly (< 10%) to the adsorption mechanism for each gas in Zn-atz-oba.

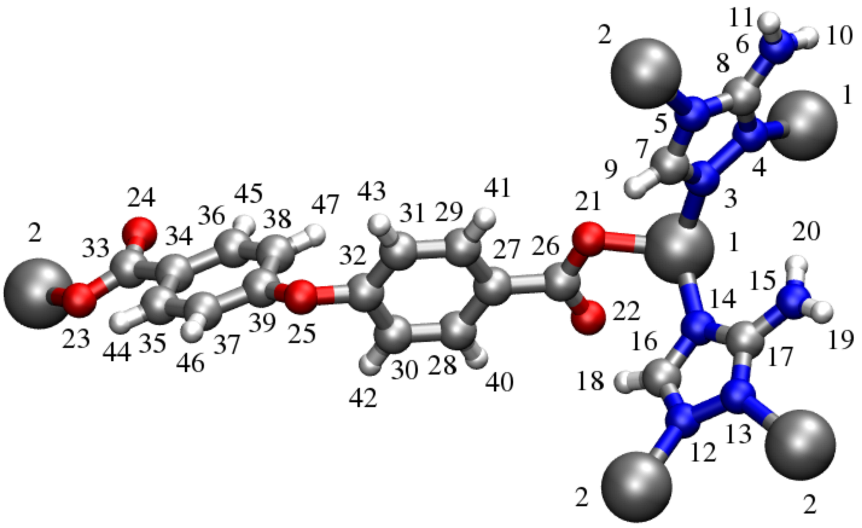

Supplementary Figure 15. The chemically distinct atoms in Zn-atz-oba defining the numbering system used in Supplementary Data 1, 2 and 3. Atom colors: C = grey, H = white, N = blue, O = red, Zn = silver.

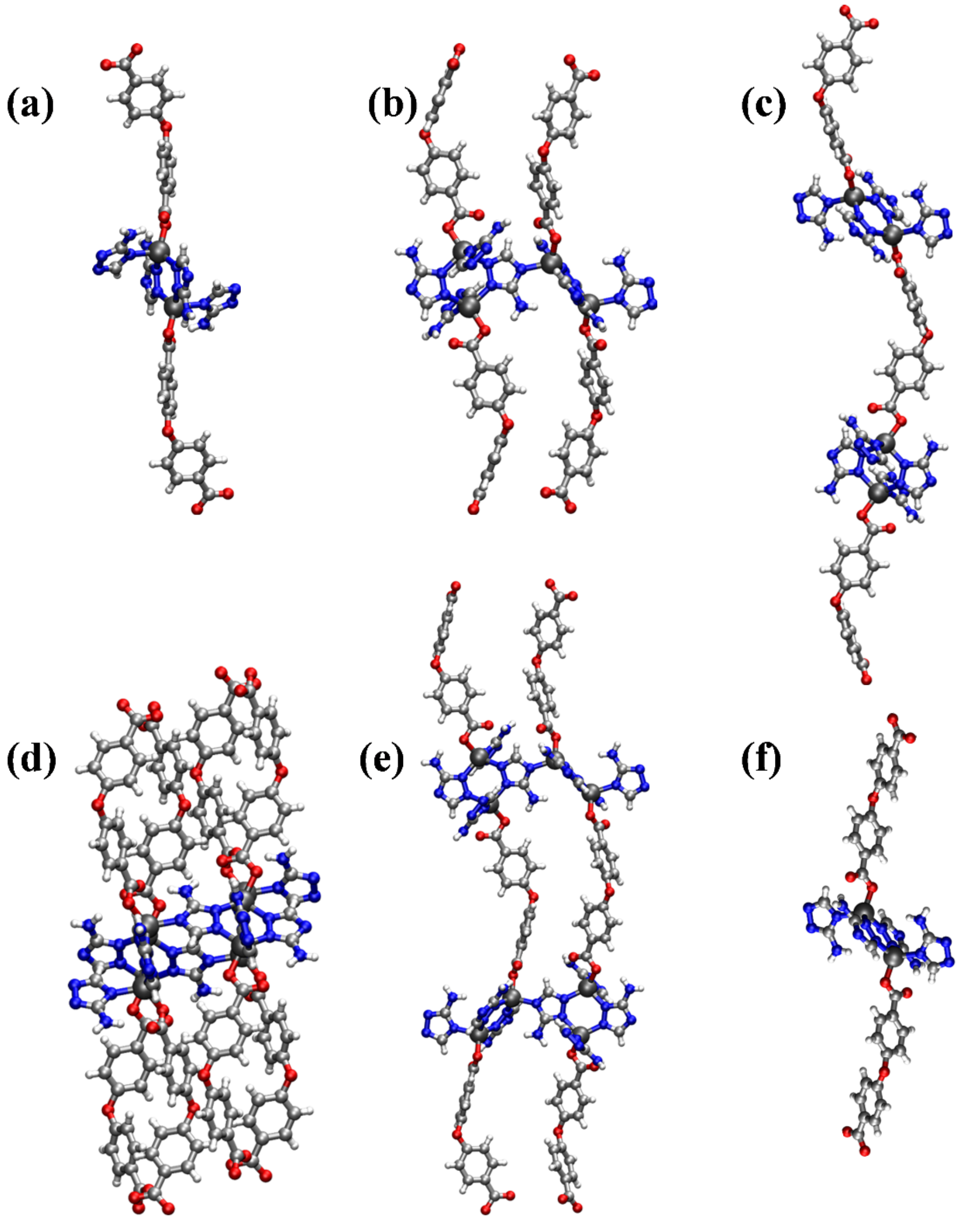

Supplementary Figure 16. Fragments of Zn-atz-oba that were selected for gas phase charge fitting calculations. Atom colors: C = cyan, H = white, N = blue, O = red, Zn = silver.

Supplementary Table 4. Calculated Q^0_{st} value (in kJ mol^{-1}) for C_2H_2, C_2H_4, C_2H_6, and CO_2 in Zn-atz-oba according to GCMC simulations at 273 K and 0.001 atm.

**Adsorbate**	**Q^0_{st} (kJ mol^{-1})**
C_2H_2	31.447
C_2H_4	31.231
C_2H_6	34.244
CO_2	32.846

Supplementary Table 5. Calculated selectivities for 50:50 mixtures of C_2H_2/C_2H_4, C_2H_6/C_2H_4, and CO_2/C_2H_4 in Zn-atz-oba at 298 K and 1 atm as determined from GCMC binary mixture simulations.

**Mixture**	**Selectivity**
C_2H_2/C_2H_4	1.0465
C_2H_6/C_2H_4	1.3446
CO_2/C_2H_4	1.1923

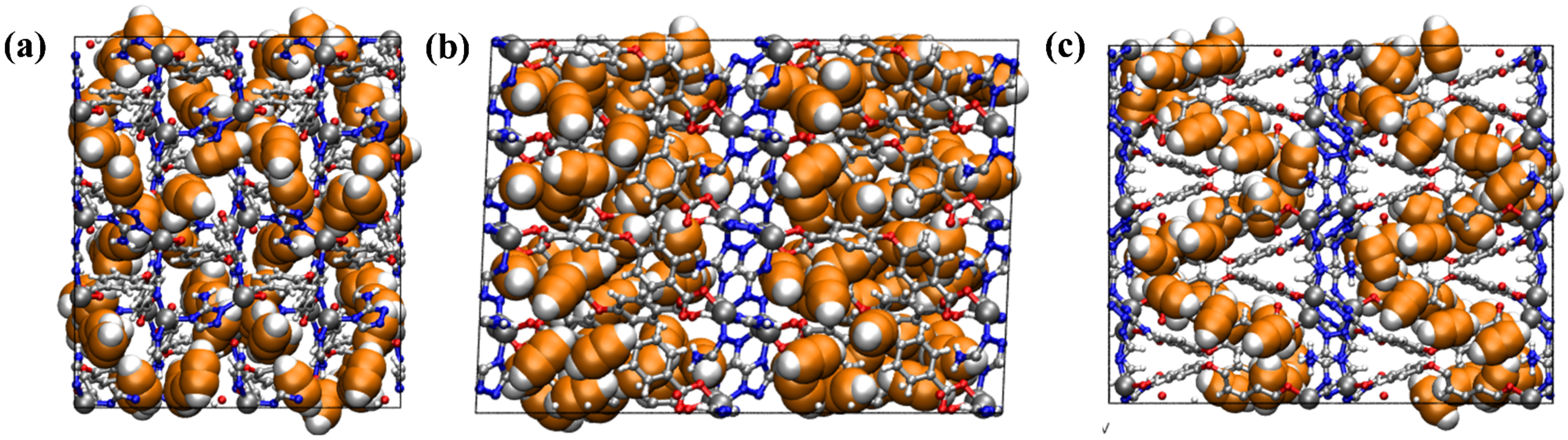

Supplementary Figure 17. (a) Orthographic *a*-axis view, (b) *b*-axis view, and (c) shifted *c*-axis view of the modeled 2 × 2 × 2 supercell of Zn-atz-oba at C_2H_2 saturation, which correspond to 13 C_2H_2 molecules per unit cell. Atom colors: C(Zn-atz-oba) = grey, C(C_2H_2) = orange, H = white, N = blue, O = red, Zn = silver.

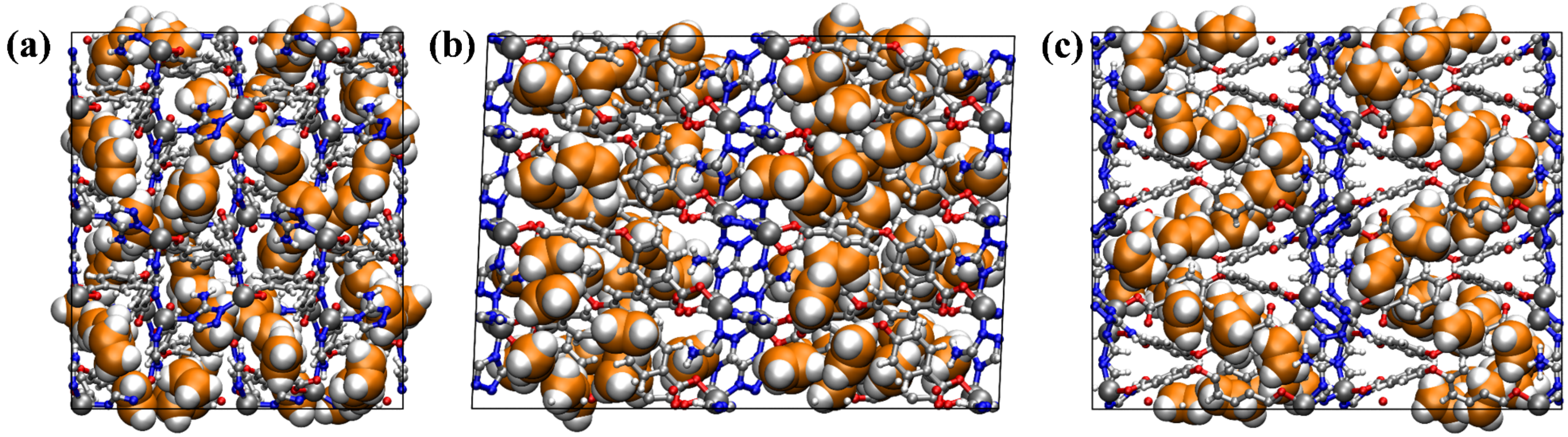

Supplementary Figure 18. (a) Orthographic *a*-axis view, (b) *b*-axis view, and (c) shifted *c*-axis view of the modeled 2 × 2 × 2 supercell of Zn-atz-oba at C_2H_4 saturation, which correspond to 11 C_2H_4 molecules per unit cell. Atom colors: C(Zn-atz-oba) = grey, C(C_2H_4) = orange, H = white, N = blue, O = red, Zn = silver.

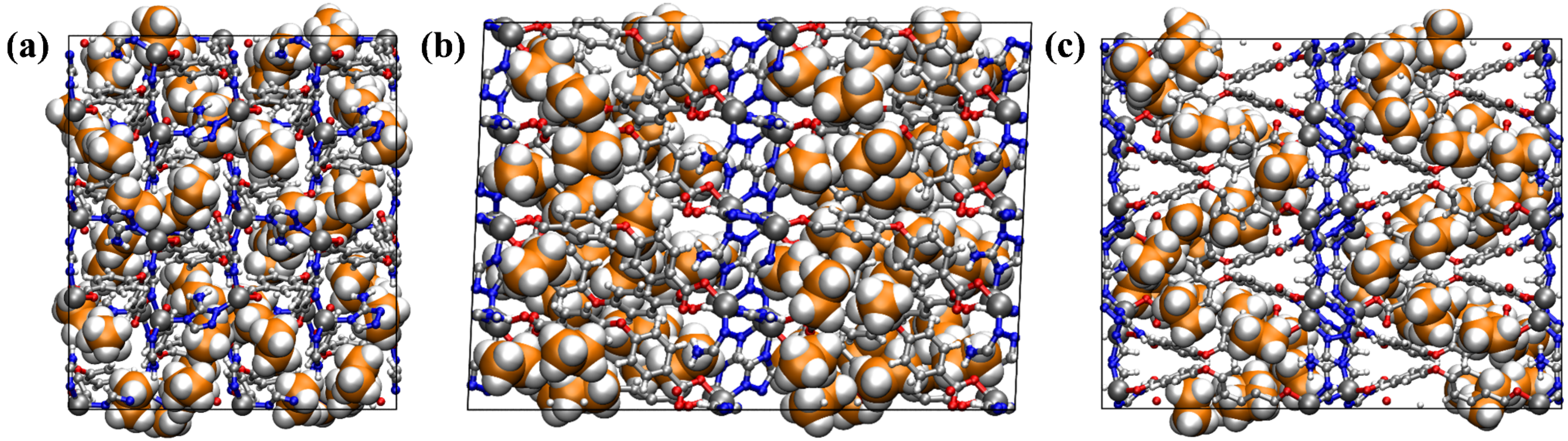

Supplementary Figure 19. (a) Orthographic *a*-axis view, (b) *b*-axis view, and (c) shifted *c*-axis view of the modeled 2 × 2 × 2 supercell of Zn-atz-oba at C_2H_6 saturation, which correspond to 10 C_2H_6 molecules per unit cell. Atom colors: C(Zn-atz-oba) = grey, C(C_2H_6) = orange, H = white, N = blue, O = red, Zn = silver.

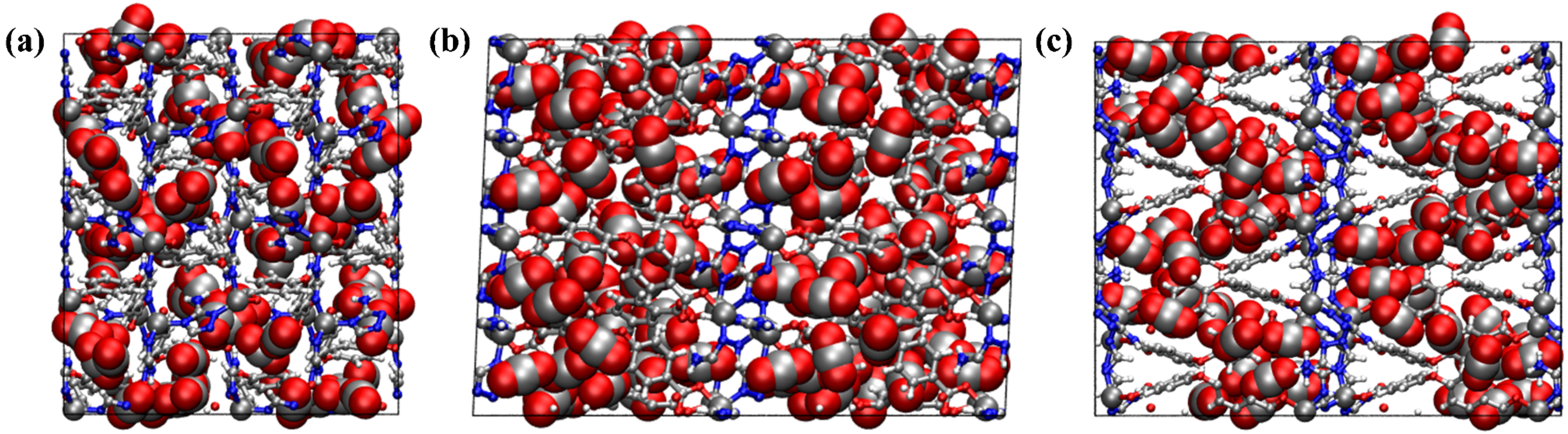

Supplementary Figure 20. (a) Orthographic *a*-axis view, (b) *b*-axis view, and (c) shifted *c*-axis view of the modeled 2 × 2 × 2 supercell of in Zn-atz-oba at CO_2 saturation, which correspond to 15 CO_2 molecules per unit cell. Atom colors: C = grey, H = white, N = blue, O = red, Zn = silver.

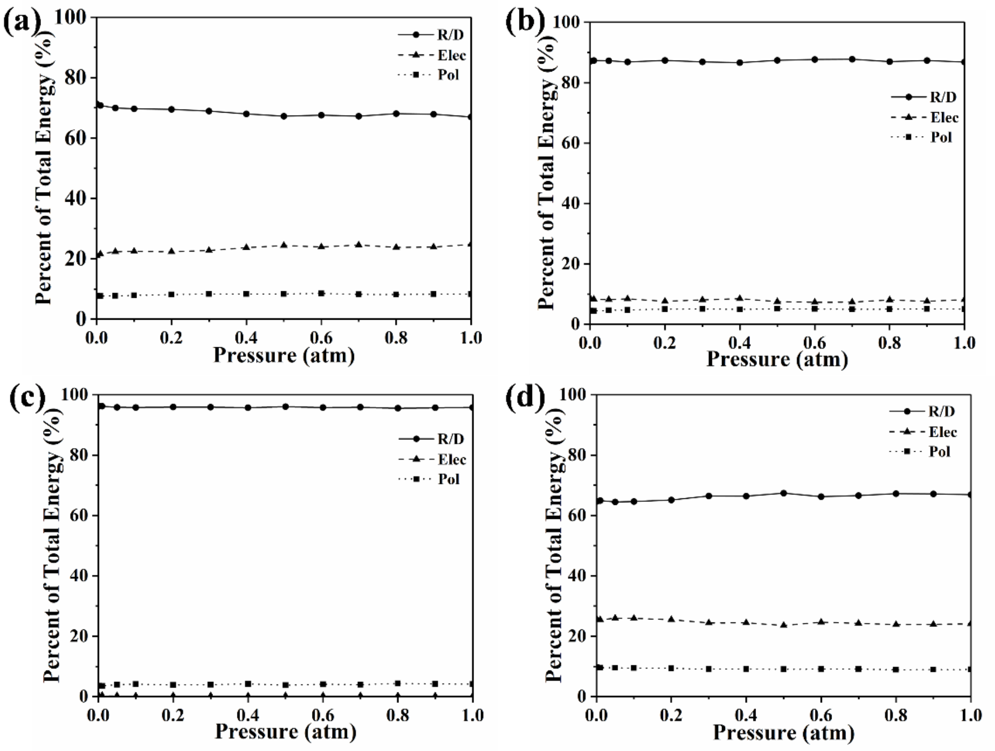

Supplementary Figure 21. Percent contributions of the energy components per adsorbate molecule for (a) C_2H_2, (b) C_2H_4, (c) C_2H_6, and (d) CO_2 in Zn-atz-oba from simulations at 298 K and pressures up to 1 atm. Line type indicates the energy component with solid lines with circles corresponding to repulsion/dispersion (R/D) contributions, dashed lines with triangles corresponding to electrostatic (Elec) contributions, and dotted lines with squares corresponding to polarization (Pol) contributions.

Dynamic gas breakthrough experiment

Productivity is derived from breakthrough experiments following this definition:

$$p = \frac{F \times y_{C2H4} \times \int_{t_1}^{t_2} \frac{C(t)}{C_0} dt}{V_m}$$

where p is the C_2H_4 productivity in mmol/g, t_1 is the C_2H_4 breakthrough time in min/g, t_2 is the breakthrough time of other gas, F is the inlet gas volume flow rate, y_{C2H4} is the volume fraction of C_2H_4 in mixed gas, C(t) is the C_2H_4 concentration in the outlet gas, C_0 is the C_2H_4 concentration in the inlet gas, V_m is molar volume of gas.

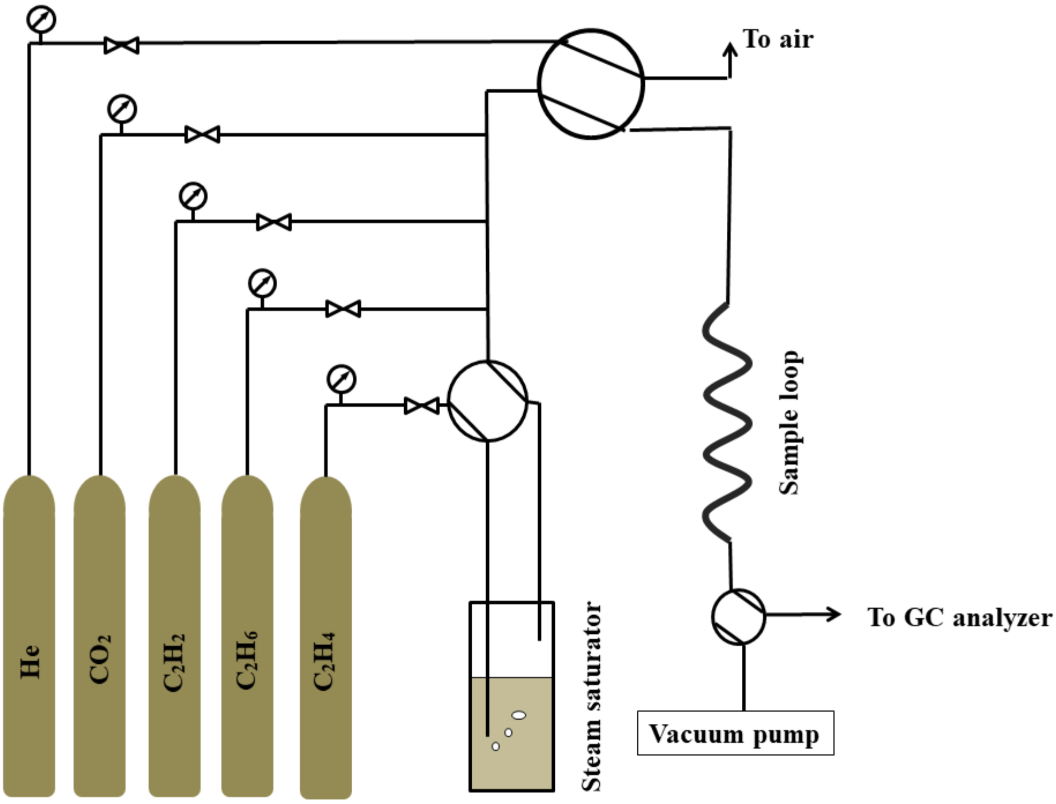

Supplementary Figure 22. Demonstration of the in-house custom-built rig used for gas breakthrough experiments.

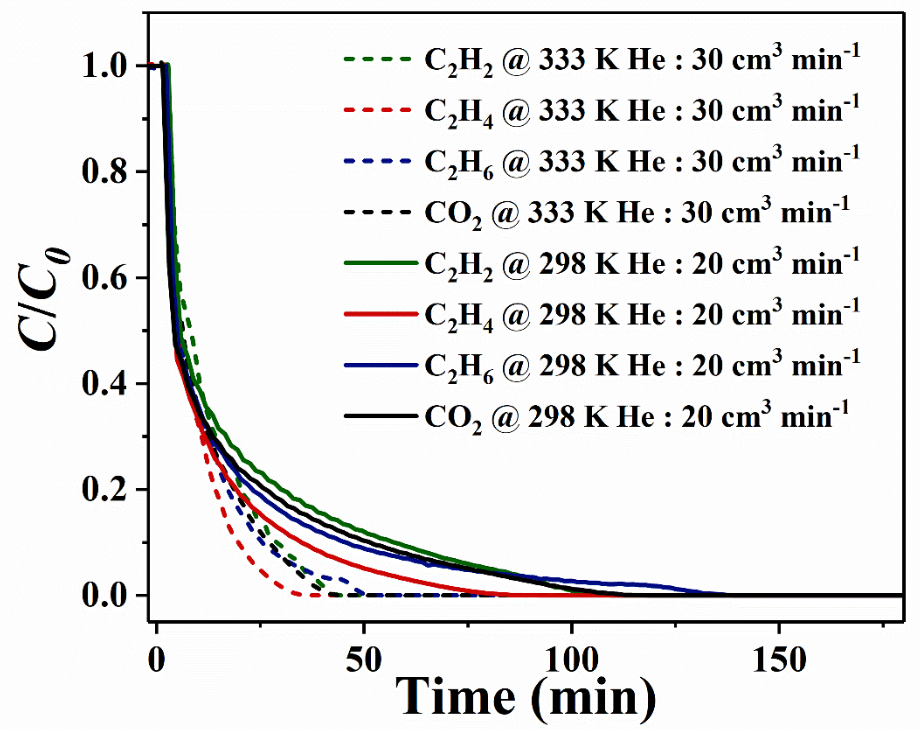

Supplementary Figure 23. Temperature-programmed desorption curves for Zn-atz-oba packed column activated under He flow of 20 $cm^3\ min^{-1}$ at 298 K (solid line) and He flow of 30 $cm^3\ min^{-1}$ at 333 K (dotted line).

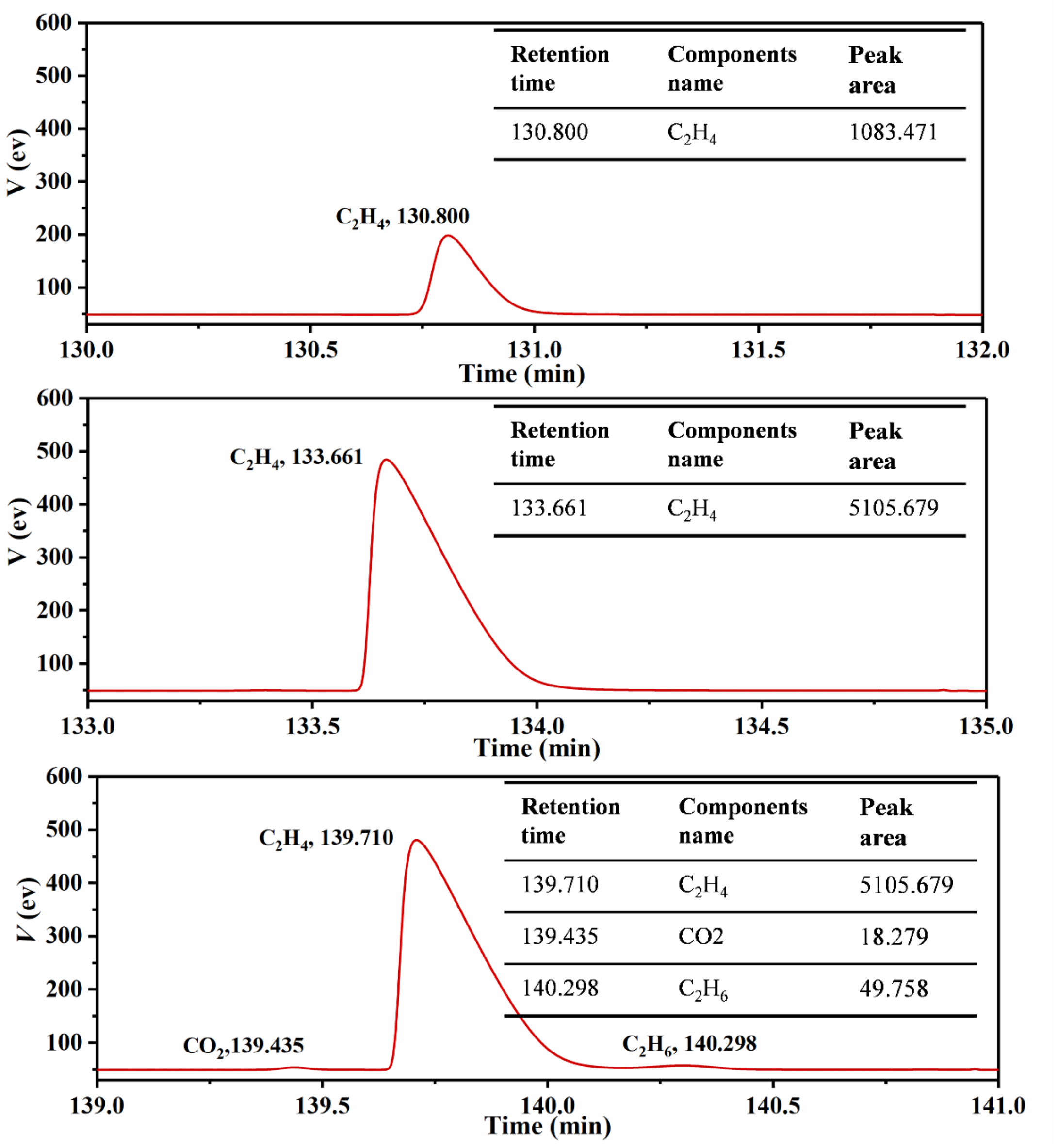

Supplementary Figure 24. Time-dependent GC analysis of the outlet gas concentration with Zn-atz-oba column (6.3 g) when flowing $C_2H_2/C_2H_4/C_2H_6/CO_2$ mixed gas (1:1:1:1 mixture; total gas pressure of 100 kPa; total gas flow of 2.8 cm^3 min^{-1}).

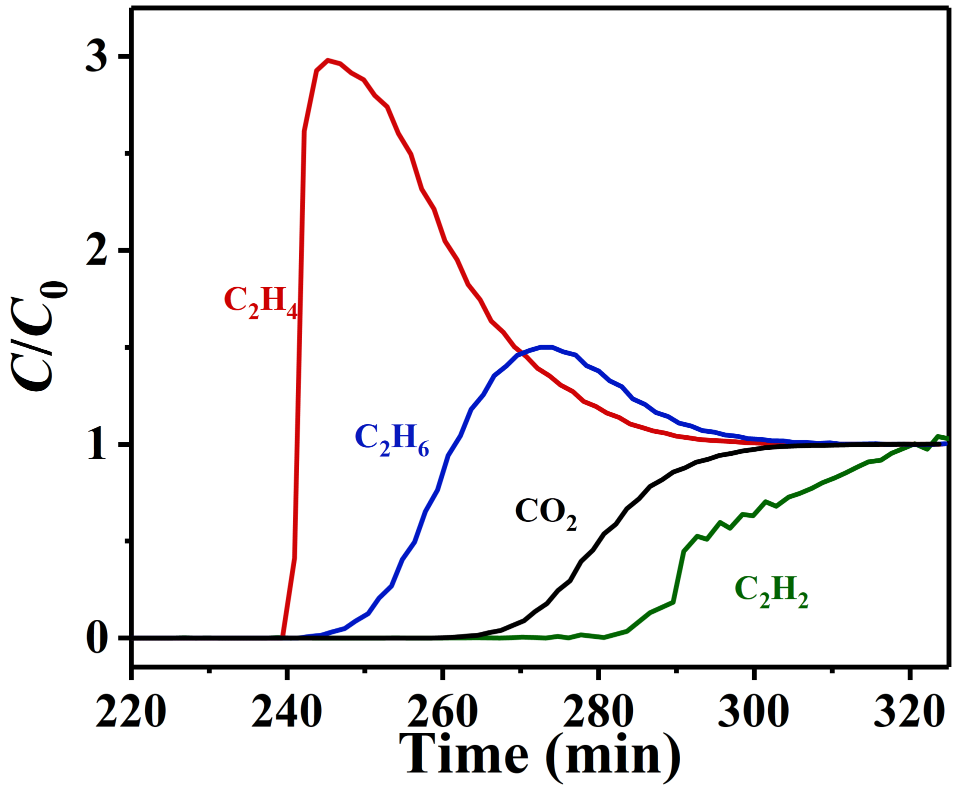

Supplementary Figure 25. Breakthrough experiment with Zn-atz-oba column (7.4 g) when $C_2H_2/C_2H_4/C_2H_6/CO_2$ mixed gas (1:33:33:33 mixture; total gas pressure of 100 kPa; total gas flow of 2.0 cm^3 min^{-1}) was flown through it.

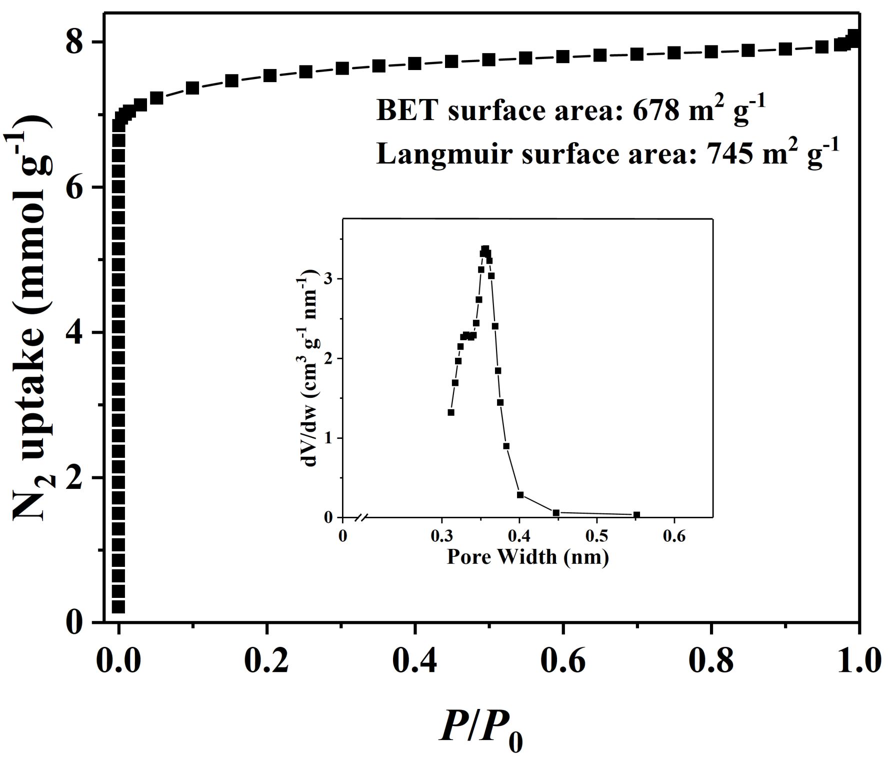

Supplementary Figure 26. N_2 isotherm at 77 K and pore distribution (H-K model, pore geometry: silt) of activated Zn-atz-oba after exposing to ambient air for 30 days.

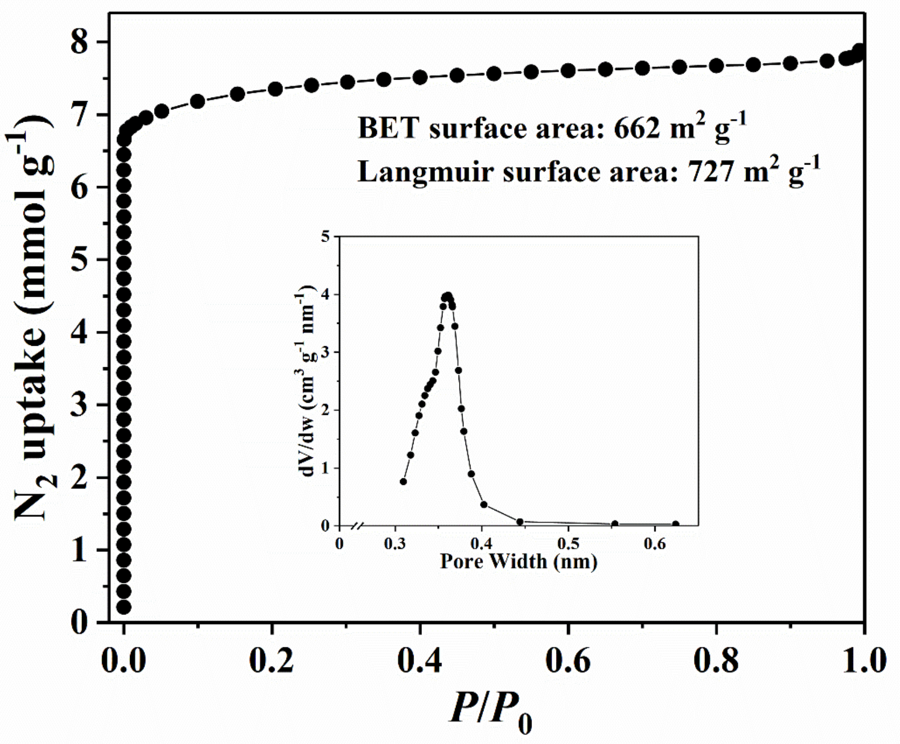

Supplementary Figure 27. N_2 isotherm at 77 K and pore distribution (H-K model, pore geometry: silt) of activated Zn-atz-oba after immersing in liquid H_2O for 10 days.

Supplementary References

1. Myers, A. L. & Prausnitz, J. M. Thermodynamics of mixed-gas adsorption. *AIChE. J.* **11**, 121-127 (1965).
2. Li, B. et al. An ideal molecular sieve for acetylene removal from ethylene with record selectivity and productivity. *Adv. Mater.* **29**, 1704210 (2017).
3. Chen, K. et al. Benchmark C_2H_2/CO_2 and CO_2/C_2H_2 separation by two closely related hybrid ultramicroporous materials. *Chem* **1**, 753-765 (2016).
4. Cui, X. et al. Pore chemistry and size control in hybrid porous materials for acetylene capture from ethylene. *Science* **353**, 141-144 (2016).
5. Yang, S. et al. Supramolecular binding and separation of hydrocarbons within a functionalized porous metal-organic framework. *Nat. Chem.* **7**, 121-129 (2015).
6. Bloch, E. D. et al. Hydrocarbon separations in a metal-organic framework with open iron (II) coordination sites. *Science* **335**, 1606-1610 (2012).
7. He, Y. et al. A microporous lanthanide-tricarboxylate framework with the potential for purification of natural gas. *Chem. Commun.* **48**, 10856 (2012).
8. Liao, P., Zhang, W., Zhang, J. & Chen, X. Efficient purification of ethene by an ethane-trapping metal-organic framework. *Nat. Commun.* **6**, 8697 (2015).
9. Chen, K. et al. Synergistic sorbent separation for one-step ethylene purification from a four-component mixture. *Science* **366**, 241-246 (2019).
10. Zeng, H. et al. Cage-Interconnected metal-organic framework with tailored apertures for efficient C_2H_6/C_2H_4 separation under humid conditions. *J. Am. Chem. Soc.* **141**, 20390-20396 (2019).
11. Qazvini, O. T., Babarao, R. & Telfer, S. G. Selective capture of carbon dioxide from hydrocarbons using a metal-organic framework. *Nat. Commun.* **12**, 197 (2021).
12. He, T., Xiao, Y., Zhao, Q., Zhou, M. & He, G. Ultramicroporous metal-organic framework Qc-5-Cu for highly selective adsorption of CO_2 from C_2H_4 stream. *Ind. Eng. Chem. Res.* **59**, 3153-3161 (2020).
13. Mukherjee, S. et al. Amino-Functionalised hybrid ultramicroporous materials that enable single-step ethylene purification from a ternary mixture. *Angew. Chem. Int. Ed.* **60**, 10902-10909 (2021).

14. Hao, H. G. et al. Simultaneous trapping of C_2H_2 and C_2H_6 from a ternary mixture of $C_2H_2/C_2H_4/C_2H_6$ in a robust metal-organic framework for the purification of C_2H_4. *Angew. Chem. Int. Ed.* **57**, 16067-16071 (2018).

15. Xu, Z. et al. A robust Th-azole framework for highly efficient purification of C_2H_4 from a $C_2H_4/C_2H_2/C_2H_6$ mixture. *Nat. Commun.* **11**, 3163 (2020).

16. Zhu, B. et al. Pore engineering for one-step ethylene purification from a three-component hydrocarbon mixture. *J. Am. Chem. Soc.* **143**, 1485-1492 (2021).

17. Wang, Y. et al. One-step ethylene purification from an acetylene/ethylene/ethane ternary mixture by cyclopentadiene cobalt-functionalized metal-organic frameworks. *Angew. Chem. Int. Ed.* **60**, 11350-11358 (2021).

18. Chen, K. et al. *Cryst. Growth Des*. New Zn-aminotriazolate-dicarboxylate frameworks: synthesis, structures, and adsorption properties. **13**, 2118-2123 (2013).

19. Jones, J. E. On the determination of molecular fields. -II. From the equation of state of a gas. *Proc. R. Soc. A* **106**, 463-477 (1924).

20. Jorgensen, W. L., Maxwell, D. S. & Tirado-Rives, J. Development and testing of the OPLS all-atom force field on conformational energetics and properties of organic liquids. *J. Am. Chem. Soc*. **118**, 11225-11236 (1996).

21. Rappé, A. K., Casewit, C. J., Colwell, K. S., Goddard, W. A. & Skiff, W. M. UFF, a full periodic table force field for molecular mechanics and molecular dynamics simulations. *J. Am. Chem. Soc*. **114**, 10024-10035 (1992).

22. Hariharan, P. C., Pople, J. A. The influence of polarization functions on molecular orbital hydrogenation energies. *Theor. Chim. Acta* **28**, 213-222 (1973).

23. Francl, M. M.et al. Self-consistent molecular orbital methods. XXIII. A polarization-type basis set for second-row elements. *J. Chem. Phys*. **77**, 3654-3665 (1982).

24. Stevens, W. J., Basch, H. & Krauss, M. Compact effective potentials and efficient shared-exponent basis sets for the first- and second-row atoms. *J. Chem. Phys*. **81**, 6026-6033 (1984).

25. Hay, P. J. & Wadt, W. R. Ab initio effective core potentials for molecular calculations. Potentials for the transition metal atoms Sc to Hg. *J. Chem. Phys*. **82**, 270-283 (1985).

26. LaJohn, L. A., Christiansen, P. A., Ross, R. B., Atashroo, T. & Ermler, W. C. Ab initio relativistic effective potentials with spin–orbit operators. III. Rb through Xe. *J. Chem. Phys*. **87**, 2812-2824 (1987).

27. Valiev, M. et al. NWChem: A comprehensive and scalable open-source solution for large scale molecular simulations. *Comput. Phys. Commun*. **181**, 1477-1489 (2010).

28. Chirlian, L. E. & Francl, M. M. Atomic charges derived from electrostatic potentials: A detailed study. *J. Comput. Chem*. **8**, 894-905 (1987).

29. Breneman, C. M. & Wiberg, K. B. Determining atom-centered monopoles from molecular electrostatic potentials. The need for high sampling density in formamide conformational analysis. *J. Comput. Chem*. **11**, 361-373 (1990).

30. Campañá, C., Mussard, B. & Woo, T. K. Electrostatic Potential Derived Atomic Charges for Periodic Systems Using a Modified Error Functional. *J. Chem. Theory Comput*. **5**, 2866–2878 (2009).

31. Manz, T. A. and Sholl, D. S. Chemically Meaningful Atomic Charges That Reproduce the Electrostatic Potential in Periodic and Nonperiodic Materials. *J. Chem. Theory Comput*. **6**, 2455–2468 (2010).

32. Wilmer, C. E., Kim, K. C. & Snurr, R. Q. An Extended Charge Equilibration Method. *J. Phys. Chem. Lett*. **3**, 2506–2511 (2012).

33. Babarao, R., Eddaoudi, M. & Jiang, J. W. Highly Porous Ionic *rht* Metal−Organic Framework for H_2 and CO_2 Storage and Separation: A Molecular Simulation Study. *Langmuir* **26**, 11196–11203 (2010).

34. Forrest, K. A. et al. Simulation of the Mechanism of Gas Sorption in a Metal–Organic Framework with Open Metal Sites: Molecular Hydrogen in PCN-61. *J. Phys. Chem. C* **116**, 15538–15549 (2012).

35. Duijnen van, P. T. & Swart, M. Molecular and atomic polarizabilities: Thole's model revisited. *J. Phys. Chem. A* **102**, 2399-2407 (1998).

36. Forrest, K. A.et al. Computational studies of CO_2 sorption and separation in an ultramicroporous metal-organic material. *J. Phys. Chem. C* **117**, 17687−17698 (2013).

37. Pham, T. et al. Understanding the H-2 sorption trends in the M-MOF-74 series (M = Mg, Ni, Co, Zn). *J. Phys. Chem. C* **119**, 1078-1090 (2015).

38. Franz, D. M. et al. Simulations of hydrogen, carbon dioxide, and small hydrocarbon sorption in a nitrogen-rich rht-metal-organic framework. *Phys. Chem. Chem. Phys*. **20**, 1761-1777 (2018).

39. Mullen, A. L. et al. A polarizable and transferable PHAST CO_2 potential for materials simulation. *J. Chem. Theory Comput*. **9**, 5421-5429 (2013).

40. Ewald, P. P. Die berechnung optischer und elektrostatischer gitterpotentiale. *Ann. Phys*. **369**, 253-287 (1921).

41. Wells, B. A. & Chaffee, A. L. Ewald summation for molecular simulations. *J. Chem. Theory Comput*. **11**, 3684-3695 (2015).

42. Applequist, J., Carl, J. R. & Fung, K.K. Atom dipole interaction model for molecular polarizability. Application to polyatomic molecules and determination of atom polarizabilities. *J. Am. Chem. Soc*. **94**, 2952-2960 (1972).

43. Thole, B. Molecular polarizabilities calculated with a modified dipole interaction *Chem. Phys*. **59**, 341-350 (1981).

44. Bode, K. A. & Applequist, J. A new optimization of atom polarizabilities in halomethanes, aldehydes, ketones, and amides by way of the atom dipole interaction model. *J. Phys. Chem*. **100**, 17820-17824 (1996).

45. McLaughlin, K., Cioce, C. R., Pham, T., Belof, J. L. & Space, B. Efficient calculation of many-body induced electrostatics in molecular systems. *J. Chem. Phys*. **139**, 184112 (2013).

46. Belof, J. L. & Space, B. *Massively Parallel Monte Carlo (MPMC)*, available on GitHub. https://github.com/mpmccode/mpmc (2012).

47. Franz, D. M. et al. MPMC and MCMD: Free high-performance simulation software for atomistic systems. *Adv. Theory Simul*. **2**, 1900113 (2019).

48. Kirkpatrick, S., Gelatt, C. D. & Vecchi, M. P. Optimization by simulated annealing. *Science* **220**, 671-680 (1983).

49. Metropolis, N., Rosenbluth, A. W., Rosenbluth, M. N., Teller, A. H. & Teller, E. Equation of state calculations by fast computing machines. *J. Chem. Phys.* **21**, 1087-1092 (1953).

50. Nicholson, D. & Parsonage, N. G. *Computer Simulation and the Statistical Mechanics of Adsorption*; Academic Press: London, pp. 97 (1982).
