# Supplementary material for: One-step ethylene production from a four-component gas mixture by a single physisorbent
Source: Nat Commun. 2021 Nov 11;12:6507. doi: 10.1038/s41467-021-26473-8 (PMC8586343; doi:10.1038/s41467-021-26473-8)
Supplement: Supplementary file 2 — Description of Additional Supplementary Files [file 41467_2021_26473_MOESM2_ESM.pdf]

Description of Additional Supplementary Files

Supplementary Data 1. Calculated average partial charges (*e*⁻) for the various chemically distinct atoms for the fragments that were selected for **Zn-atz-oba**. The average and standard deviation between the fragments are also included. Labeling of atoms and fragments correspond to **Supplementary Figures 15** and **16**, respectively.

Supplementary Data 2. Parameters for the chemically distinct atoms in **Zn-atz-oba** that were used for the simulations. Atom labels correspond to the **Supplementary Figure 15**.

Supplementary Data 3. The crystallographic distances (in Å) between various atoms in **Zn-atz-oba**. Atom labels correspond to the **Supplementary Figure 15**.
